# Apical Cell-Cell Adhesions Reconcile Symmetry and Asymmetry in Zebrafish Neurulation

**DOI:** 10.1016/j.isci.2018.04.007

**Published:** 2018-04-13

**Authors:** Chuanyu Guo, Jian Zou, Yi Wen, Wei Fang, Donna Beer Stolz, Ming Sun, Xiangyun Wei

**Affiliations:** 1 Department of Ophthalmology, University of Pittsburgh, 3501 Fifth Avenue, Pittsburgh, PA 15213, USA; 2 Department of Cell and Physiology, University of Pittsburgh, 3501 Fifth Avenue, Pittsburgh, PA 15213, USA; 3 Department of Developmental Biology, University of Pittsburgh, School of Medicine, 3501 Fifth Avenue, Pittsburgh, PA 15213, USA; 4 Department of Microbiology and Molecular Genetics, University of Pittsburgh, School of Medicine, 3501 Fifth Avenue, Pittsburgh, PA 15213, USA

**Keywords:** Ichthyology, Developmental Neuroscience, Evolutionary Developmental Biology

## Abstract

The symmetric tissue and body plans of animals are paradoxically constructed with asymmetric cells. To understand how the yin-yang duality of symmetry and asymmetry are reconciled, we asked whether apical polarity proteins orchestrate the development of the mirror-symmetric zebrafish neural tube by hierarchically modulating apical cell-cell adhesions. We found that apical polarity proteins localize by a pioneer-intermediate-terminal order. Pioneer proteins establish the mirror symmetry of the neural rod by initiating two distinct types of apical adhesions: the parallel apical adhesions (PAAs) cohere cells of parallel orientation and the novel opposing apical adhesions (OAAs) cohere cells of opposing orientation. Subsequently, the intermediate proteins selectively augment the PAAs when the OAAs dissolve by endocytosis. Finally, terminal proteins are required to inflate the neural tube by generating osmotic pressure. Our findings suggest a general mechanism to construct mirror-symmetric tissues: tissue symmetry can be established by organizing asymmetric cells opposingly via adhesions.

## Highlights

• Apical polarity proteins localize in a pioneer-intermediate-terminal order
• The orderly localized proteins orchestrate apical adhesion dynamics step by step
• Apical adhesions assemble asymmetric cells opposingly into a symmetric tissue

## Graphical Abstract

**Figure:**
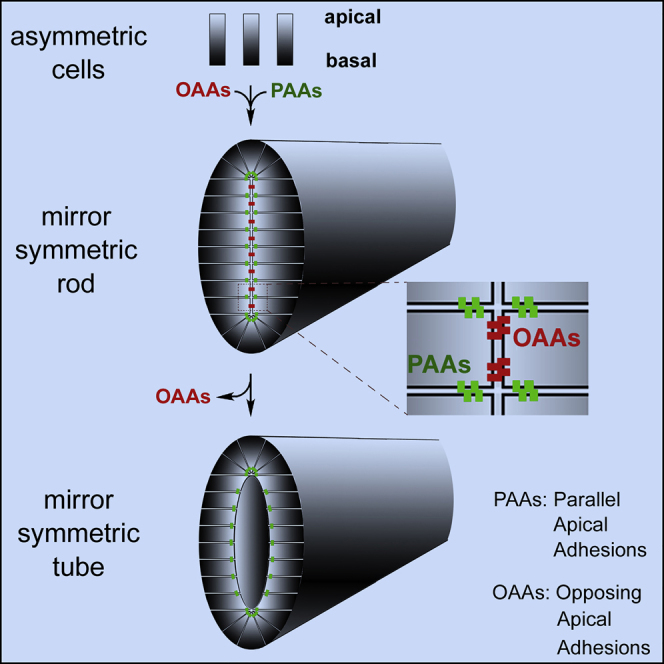

## Introduction

Symmetry is a hallmark of metazoan body plans as well as tissue and organ architectures (Martindale et al., 2002). However, the cellular building blocks of metazoans are asymmetric. Thus arises the fundamental biological and even philosophical question of just how the yin-yang duality of asymmetry and symmetry is reconciled during tissue morphogenesis. This general question may be addressed by studying vertebrate neurulation because this morphogenesis robustly builds a mirror-symmetric neural tube *de novo* from asymmetrically polarized neuroepithelial cells through opposing configuration.

Vertebrate neurulation occurs via either the “epithelium-wrapping” mode, in which an epithelial sheet wraps around pre-existing apical extracellular space as an interior lumen, or the “lumen-inflation” mode, in which epithelial cells aggregate to first form a solid rod from which an interior lumen subsequently emerges (Davidson and Keller, 1999, Colas and Schoenwolf, 2001, Lowery and Sive, 2004). Proper neurulation by either mode requires a delicate balance between cell plasticity and cell cohesiveness. On the one hand, cells need to be plastic to reorganize their relative positions and to modify their shapes to form a tube; on the other hand, cells need to be cohesive with each other to maintain certain tissue architectures. The key to a delicate balance between plasticity and cohesiveness is the modulation of cell-cell adhesion. This is because excessive cell-cell adhesion would compromise cell plasticity and insufficient cell-cell adhesion would compromise cell cohesiveness. In epithelia, an important component of cell-cell adhesion is the apical adhesions, including the classic tight junctions (TJs) and adherens junctions (AJs); these apical adhesions are maintained and regulated by many apical polarity proteins (Harris and Tepass, 2010, Pocha and Knust, 2013). Thus apical polarity proteins must dynamically modulate apical adhesions, which in turn regulate cellular reorganization during vertebrate neurulation.

Supporting this notion, mutations that disable various apical polarity proteins affect zebrafish neurulation, which utilizes the “lumen-inflation” mode and sequentially undergoes the neural plate, neural keel, neural rod, and finally neural tube stages (Tawk et al., 2007). Although many apical proteins are required for proper neurulation, their loss-of-function phenotypes vary drastically, particularly in the timing of phenotypic manifestation. For example, *N-cadherin* (*N-Cad*) mutants display defects starting at the neural keel stages (Lele et al., 2002, Malicki et al., 2003, Chalasani and Brewster, 2011), whereas the *Nagie oko*^*m520*^ (*nok* ^*m520*^) mutants, in which the homolog of mammalian *pals1* and *Drosophila stardust* genes (Kamberov et al., 2000, Hong et al., 2001) is disabled, do not show defects until the neural tube stages (Wei and Malicki, 2002).

These phenotypic variations suggest a temporal hierarchy of apical polarity proteins in regulating neurulation. Supporting this notion, we previously found that a supernumerary neural tube defect can be introduced by precocious expression of Lin7c, whose apical localization normally lags behind the TJ protein ZO-1 (Yang et al., 2009). Therefore we hypothesize that during zebrafish neurulation, apical polarity proteins localize in a strict spatiotemporal order and dynamically regulate apical cell-cell adhesions so as to cohere asymmetric neuroepithelial cells opposingly into the mirror-symmetric neural rod and neural tube; by regulating both the asymmetry property of individual cells and the mirror symmetry property of the tissue, apical adhesions reconcile asymmetry and symmetry, a yin-yang duality per the ancient Chinese philosophy of Daoism because they are seemingly contradicting and yet inseparable and interchanging. Thus the coordination of asymmetry and symmetry during neurulation may reflect a principle that has broader applications.

To test the above-mentioned hypothesis, we chose to study various apical polarity proteins that are representatives for the conventional TJs and AJs; we also studied components of the Crumbs and Par3 apical polarity protein complexes, which regulate TJs and AJs (Pocha and Knust, 2013, Harris and Tepass, 2010). With genetic, molecular, and imaging approaches (see Transparent Methods in Supplemental Information), we analyzed the spatiotemporal order of their localizations and their roles in regulating two types of apical adhesions: the parallel apical adhesions (PAAs), which cohere cells of parallel orientation, and the novel opposing apical adhesions (OAAs), which cohere cells of opposing orientation. Our findings confirmed our hypothesis and established a three-step spatiotemporal framework by which apical polarity proteins regulate zebrafish neurulation. Moreover, our study suggests a general mechanism by which asymmetric cells organize into mirror-symmetric tissues.

## Results

### Hierarchical Localization of Apical Polarity Proteins and the Dynamics of Apical Surfaces

Of the many apical polarity proteins, we chose to examine N-Cad, E-Cad, ZO-1, β-catenin, F-actin bundles, Crumbs 1(Crb1), Crb2a, Nok, aPKC, Pard3, and Na^+^/K^+^-ATPase α. Although these proteins account for only a fraction of the known apical polarity proteins, they are good representatives because they constitute and regulate the TJs and AJs and regulate the extracellular osmotic pressure that inflates the neural tube lumen. Furthermore, genetic evidence has shown that they or their partners regulate zebrafish neurulation: N-Cad (Lele et al., 2002, Malicki et al., 2003), Claudins (Zhang et al., 2012), Pard3 (Wei et al., 2004), aPKC (Horne-Badovinac et al., 2001), Pard6 (Munson et al., 2008), Crumbs (Crb) (Malicki and Driever, 1999), Nok (Wei and Malicki, 2002), Lin7c (Wei et al., 2006, Yang et al., 2009), and Na^+^/K^+^-ATPase (Lowery and Sive, 2005, Chang et al., 2012).

To accurately define the spatiotemporal order of the apical localization of these polarity proteins, we examined their distribution in the hindbrain's rhombomeres 5 and 6 by simultaneously visualizing two or more proteins under confocal microscopy. Rhombomeres 5 and 6 were chosen because these regions can be easily identified by the adjacent landmarks, the otic vesicles (Moens et al., 1998, Barald and Kelley, 2004).

**Figure 1:**
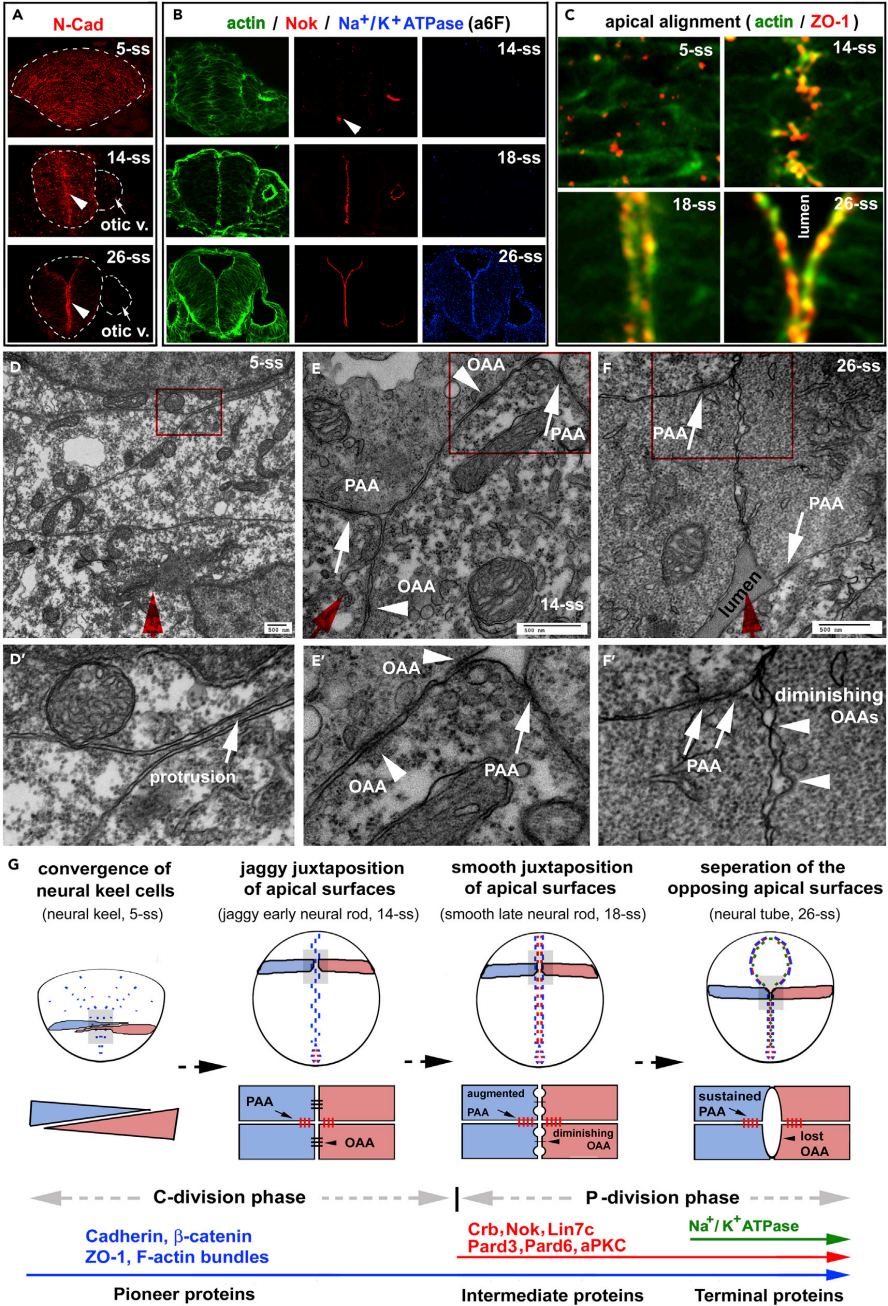
Three-Step Localizations of Apical Polarity Proteins Correlate with the Dynamics of Apical Cell-Cell Adhesions during Zebrafish Neurulation (A) Pioneer proteins N-Cad, visualized by immunohistochemistry, distributed ubiquitously on the cell membranes at 5-ss and then enriched apically (arrowheads) at 14-ss and 26-ss. Arrows, the otic vesicle. (B) Simultaneous staining of F-actin bundles, Nok, and Na^+^/K^+^ ATPase at 14-ss, 18-ss, and 26-ss. Note the lack of Nok signals in the neural tissue at 14-ss, except at the ventral end (arrowhead), and the lack of Na^+^/K^+^ ATPase apical enrichment at 14-ss and 18-ss. (C) The F-actin bundles and ZO-1 (in the gray areas in G) scattered in the neural keel (5-ss), aligned jaggedly in the early neural rod (14-ss), and aligned smoothly in the late neural rod (18-ss) and the neural tube (26-ss). (D–F and D′–F′) TEM revealed the dynamics of the PAAs and OAAs at the midline region during neurulation: no apparent electron-dense cell-cell junctional complexes in the neural keel at 5-ss (D and D′); apparent electron-dense PAAs (white arrows) and OAAs (white arrowheads) in the jaggy early neural rod at 14-ss (E and E′); and diminishing OAAs and persistent PAAs in the smooth late rod at 18-ss or the neural tube region where apical surfaces still juxtaposed at 26-ss (F and F′). D′–F′ are magnifications of the local regions boxed in D–F, respectively. The red arrows indicate the midline axis, which was defined as the dorsal-ventral central axis of the cross sections of the entire tissues under low magnifications. (G) Diagrams summarize the spatiotemporal localization order of pioneer, intermediate, and terminal proteins; the morphological changes of apical surface alignments; the dynamics of the PAAs and OAAs,; and the switch from cross-midline cell division mode (C-division) to parallel cell division mode (P-division) during the “neural keel-jaggy early neural rod-smooth late neural rod-neural tube” transition. Blue and magenta illustrate neuroepithelial cells of opposite orientations. Also see Figure S1.

We found that the localization of apical polarity proteins follows a strict three-step spatiotemporal order; thus we categorize these proteins accordingly into three groups: “pioneer,” “intermediate,” and “terminal” proteins. The pioneer proteins, including E-Cad, N-Cad, β-catenin, ZO-1, and F-actin bundles, generally first localize to the entire cell membranes at the neural keel stages (5-somite stage, or 5-ss for short), then enrich apically at the early neural rod stages (around 14-ss), and afterward persist at the apical regions (Figures 1A–1C, 1G, and S1). However, there are two exceptions to this general dynamic. First, ZO-1 never distributes to the entire membrane; rather, it emerges initially as punctate spots at the neural keel stages (Figure S1A). Second, the E-Cad level at the apical surface, particularly at the ventral region, is reduced at the neural tube stages (Figure S1A).

Unlike the pioneer proteins, the intermediate proteins, including Crb1, Crb2a, Nok, Lin7c, Pard3, and aPKC, never localized to the entire cell membrane; rather, they emerged apically at the late neural rod stage (around 18-ss) after the pioneer proteins had already localized apically and after neuroepithelial cell proliferation had switched from cross-midline cell division (C-division) mode to parallel-midline cell division (P-division) mode (Figures 1B and 1G, and S1B–S1F; Yang et al., 2009). The finding that Pard3 localized apically after C-division challenges a previous claim, made by expressing the Pard3-green fluorescent protein (GFP) through messenger RNA (mRNA) injection, that Pard3 is required for C-division and for establishing the mirror symmetry of the neural rod or the neural tube (Tawk et al., 2007). Finally, at the onset of lumen inflation, the terminal apical polarity protein Na^+^/K^+^-ATPase α enriched at the apical regions, with a much weaker presence on the lateral membranes (Figures 1B, 1G, S1B, and S1C) (this apical enrichment of Na^+^/K^+^-ATPase α was also shown previously; Lowery and Sive, 2005). Across the neural tissue, this three-step temporal localization order does not occur simultaneously everywhere; instead, the apical localization and enrichment occur earlier in the ventral and anterior regions than in the dorsal and posterior regions (Figure 1B, arrowhead; Yang et al., 2009).

The progress of the three-step localization of apical polarity proteins is correlated with the changing midline alignments of the apical cell surfaces, as highlighted with apical marker ZO-1 and F-actin bundles. First, when the pioneer proteins become enriched apically at the early stages of the neural rod (∼14-ss), the apical surfaces align jaggedly around the midline (Figures 1C and 1G); we thus name the neural rod at this stage “the jaggy early neural rod,” or “the early neural rod” for short. Second, when the intermediate proteins localize apically, the apical surfaces become smoothly aligned and the ZO-1 sites are segregated into two parallel planes flanking the midline (Figures 1C and 1G); we thus name the neural rod at this stage “the smooth late neural rod,” or “the late neural rod” for short. Third, when the terminal proteins enrich at the apical surfaces, the left and right apical surfaces start to separate from each other by the emerging lumen (Figures 1C and 1G). These morphological changes of apical cell surfaces and the hierarchical localization of apical proteins imply that cell-cell adhesions change dynamically at the apical regions because apical polarity proteins directly or indirectly regulate cell-cell adhesions (Knust, 2002; Harris and Tepass, 2010). Thus a revelation of the dynamics of the apical cell-cell adhesions becomes critical in understanding neurulation.

### Two Distinct Types of Apical Adhesions during Neurulation: the PAAs and the OAAs

We, thus, next examined under transmission electron microscopy (TEM) the apical cell-cell adhesions at different developmental stages. At the neural keel stages, before pioneer proteins enrich apically (∼5-ss), no typical electron-dense cell-cell adhesion complexes appeared at the apical ends of the cells; furthermore, the apical ends of over 50% of cells (42 cells in two embryos) protruded into the opposing half of the tissue, far beyond the midline (Figures 1D, 1D′, and 1G). By contrast, at 14-ss, the jaggy early neural rod stage, pioneer proteins localized apically, the apical surfaces of the cells were aligned in a jaggy fashion around the midline (Figure 1C), and no long apical protrusions crossing the midline were observed (67 cells in two embryos; Figures 1E, 1E′, 1G, and S1G). At this stage, we observed two different types of electron-dense adhesion complexes that aligned as a single jaggy line at the midline. One was formed between cells of parallel apicobasal orientation, hereafter named the PAAs, which encompass the AJs and TJs, and were present in all neuroepithelial cells. The other, a novel type of adhesion complex, was formed between cells of opposite apicobasal orientations at a frequency of 1–2 adhesions per cell per TEM section (79 cells in 15 TEM sections of three embryos); we named these adhesions the OAAs (Figures 1E, 1E′, and 1G). We name these adhesions the PAAs and OAAs for two reasons: first, to highlight the orientation differences between the adhered cells and second, to imply that the composition of these adhesions undergo developmental changes, and as a result, they may not be simply regarded as the AJs and TJs in the conventional sense.

Subsequently, at the smooth late neural rod stages (around 18-ss), after intermediate proteins localize to the apical surface, the apical surfaces were smoothly aligned to flank the midline. At this stage, the PAAs remained prominent and the left and right PAAs aligned in two closely positioned but segregated vertical planes; by contrast, the OAAs became diminishing, and the opposing apical cell membranes became segregated by small fragmented lumens (Figures 1F, 1F′, and 1G). Eventually, the OAAs dissolved at the neural tube stages, and the opposing apical cell membranes separated completely and a continuous lumen emerged.

In summary, the PAAs *permanently* hold the cells together *within* the left and right halves of the tissue from the early neural rod stages onward (of course, at the roof plate and the floor plate, the PAAs are still required to hold cells together *between* the left and right halves). By contrast, the OAAs *transiently* join the cells *between* the left and right halves at the midline in opposing orientations at the early neural rod stages.

### The Compositions and Dynamics of the PAAs and OAAs

The PAAs and OAAs emerge when pioneer proteins enrich apically and the OAAs dissolve when the intermediate proteins localize apically (Figure 1G). These coincidences raise an interesting question: How do pioneer and intermediate apical polarity proteins correlate with the compositions and developmental dynamics of the PAAs and OAAs?

**Figure 2:**
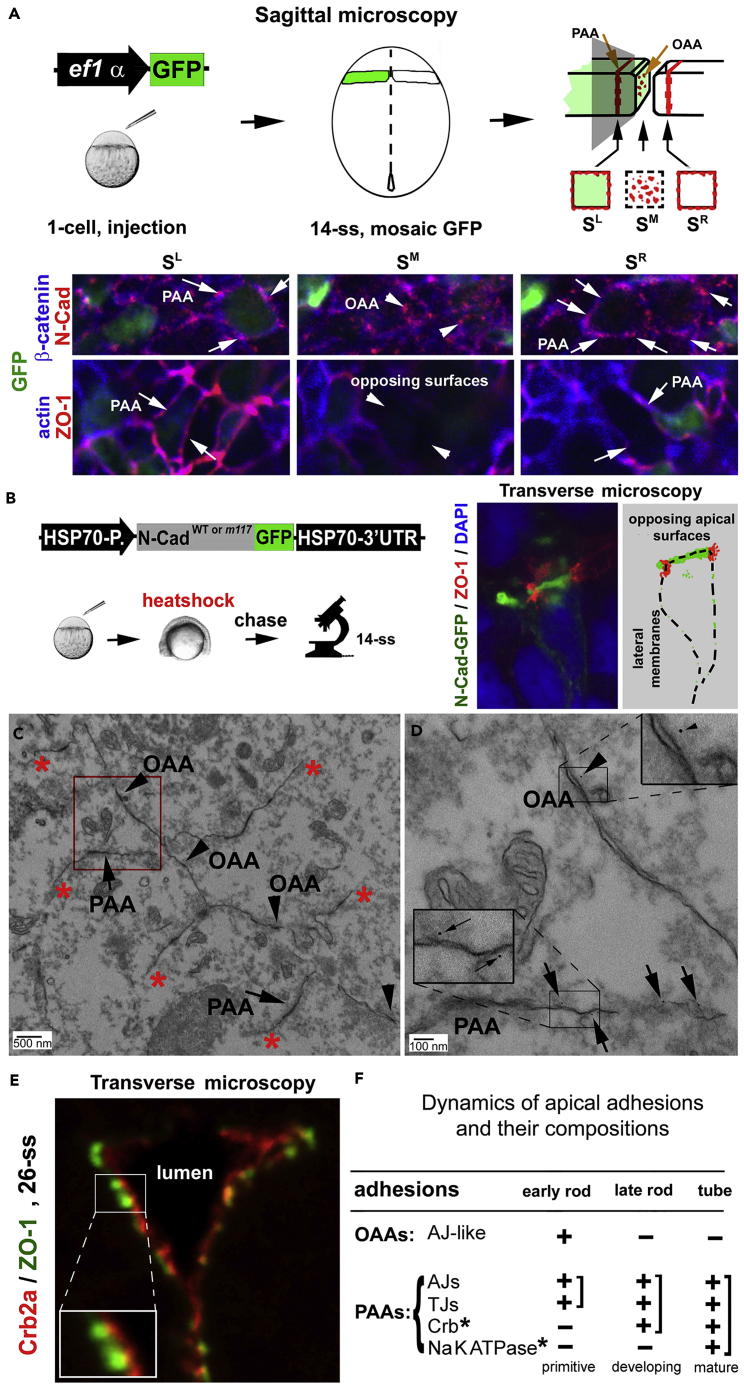
Dynamics and Compositions of the PAAs and OAAs (A) Diagrams illustrate the principle of GFP-assisted sagittal serial microscopy for visualizing proteins at the opposing apical surfaces: the opposing apical surfaces at the midline region (S^M^), which contain the OAAs, must be flanked by two spatial references—PAA regions of the left and right halves of the tissue (S^L^ and S^R^). Green represents transiently expressed GFP in some cells. The bottom panels are images collected with this technique: N-Cad and β-catenin were detected at the PAA regions as circumferential belts (S^L^ and S^R^, arrows) as well as in the OAA regions as punctate foci (S^M^; arrowheads). By contrast, ZO-1 and actin were detected in the PAA regions (S^L^ and S^R^, arrows) but not in the OAA regions (S^M^, arrowheads). (B) Diagrams illustrate the strategy to visualize transiently expressed N-Cad^wt^-GFP or N-Cad^m117^-GFP at 14-ss. Note that N-Cad^wt^-GFP enriched on the opposing apical surfaces (illustrated by the drawing on the right). (C) The PAAs (arrows) and the opposing apical surfaces (arrowheads indicating the OAAs) survived the Triton X-100 extraction of the anti-GFP immunogold electron microscopic procedure, whereas the lateral membranes were dissolved (basal of asterisks). (D) The magnification of the boxed region in C revealed the labeling of N-Cad-GFP with 5-nm gold particles at the PAAs (arrows, 72% of the PAAs were labeled with gold particles) and OAAs (arrowhead). Insets are magnifications of boxed regions. (E) Crb2a juxtaposed with ZO-1 sites at the apical ends (26-ss). (F) The table summarizes the developmental and compositional changes of the OAAs and PAAs. Crb*, Crb-based adhesion; NaKATPase*, Na^+^/K^+^-ATPase-based adhesion; +, presence; -, absence. Also see Figure S2.

We thus closely examined the *en face* distributions of pioneer proteins N-Cad, ZO-1, β-catenin, and actin bundles at the apical regions of neuroepithelial cells at 14-ss with GFP-labeling-assisted serial sagittal immunomicroscopy (Figure 2A). We found that at opposing apical surfaces, N-Cad and its cytoplasmic partner β-catenin localized in punctate foci, which presumably represent the OAAs (Figure 2A, S^M^, arrowheads; Figure S2A). The localization of N-Cad to the opposing apical surfaces was next verified by the presence of transiently expressed N-Cad^wt^-GFP fusion protein (Jontes et al., 2004) (Figures 2B and S2B–S2D). In contrast, at the PAA regions, N-Cad and β-catenin distributed as circumferential belts. Unlike N-Cad, ZO-1 localized only to PAA regions as circumferential belts, which were also smoother than the N-Cad circumferential belts (Figures 2A and S2A). The localization of N-Cad to the PAAs and OAAs was further confirmed by immunoelectron microscopic labeling of N-Cad-GFP with an anti-GFP antibody, showing the enrichment of gold particles at the PAAs and OAAs in wild-type embryos that expressed N-Cad^WT^-GFP but not in non-transgenic wild-type fish (Figures 2C, 2D, S2G, and S2H). Interestingly, the PAAs and the opposing apical membranes were more resilient to detergent extraction than the lateral membrane (Figures 2C, 2D, S2G, and S2H), suggesting that these regions were more stable, supposedly due to enriched cell-cell adhesion complexes.

We next examined the distribution of intermediate proteins at the late neural rod and neural tube stages (18-ss and 26-ss, respectively). We found that these proteins were closely associated with the apical edge of ZO-1 foci (Figures 2E, S1B–S1F, and S2E). For example, at the luminal apical surface where individual PAAs can be better distinguished at 26-ss, 96% of ZO-1 foci (N = 26) associated with Crb2a at their apical edges and 70% of Crb2a foci (N = 36) associated with ZO-1 at their basal edges. Later, at neural tube stages (26-ss), terminal proteins Na^+^/K^+^-ATPase α enriched to the same apical regions where intermediate proteins Nok, aPKC, and Crb1 localized (Figure 1B, S1B, and S1C); higher magnification revealed that all Na^+^/K^+^-ATPase foci contained intermediate proteins aPKC (N = 110) and Nok (N = 105), although not all intermediate protein foci displayed significant Na^+^/K^+^-ATPase (Figure S2F).

Together, these observations suggest that the development and molecular compositions of the PAAs and OAAs are dynamic (Figure 2F). (1) The OAAs emerge during the transition from the neural keel to the early neural rod stages and dissolve at the late neural rod stages. The OAAs are punctate AJ-like junctions because they contain AJ proteins N-Cad and β-catenin but not TJ protein ZO-1 (Tsukita et al., 1992, Niessen and Gottardi, 2008); however, the complete composition of the OAAs is yet to be discovered. (2) The PAAs first emerge along with the OAAs at the early neural rod stage and initially encompass only the AJs and TJs; we thus name these PAAs the primitive PAAs. At late neural rod stages, the PAAs are joined at their apical edges by intermediate proteins, among which the Crb proteins directly mediate cell-cell adhesion with their extracellular domains, thus referred to as Crb-based adhesion (Zou et al., 2012; Figure 2F); we thus name these PAAs the developing PAAs. At the neural tube stages, the PAAs are joined by the terminal proteins, among which Na^+^/K^+^-ATPase also mediates cell-cell adhesion with their β subunit's homophilic trans-adhesion capability, which is referred to as Na^+^/K^+^-ATPase-based adhesion (Cereijido et al., 2012, Vagin et al., 2012; Figure 2F); we thus name these PAAs the mature PAAs.

### Roles of Pioneer and Intermediate Proteins in the Mirror Symmetry of the Neural Tissue

At the tissue architectural level, the coincidence between the apical enrichment of the pioneer proteins and the formation of mirror-symmetric early neural rod made us wonder whether the pioneer proteins initiate the mirror symmetry, which is subsequently maintained by the intermediate proteins. To test this hypothesis, we next assessed the effects of loss-of-function mutations of N-Cad, Nok, and Pard6γb on mirror symmetry development at 26-ss and 34 hpf (hours post fertilization) (∼12 hr after 26-ss) by examining the distribution of apical marker ZO-1 and basal marker GFAP as well as lumen formation.

**Figure 3:**
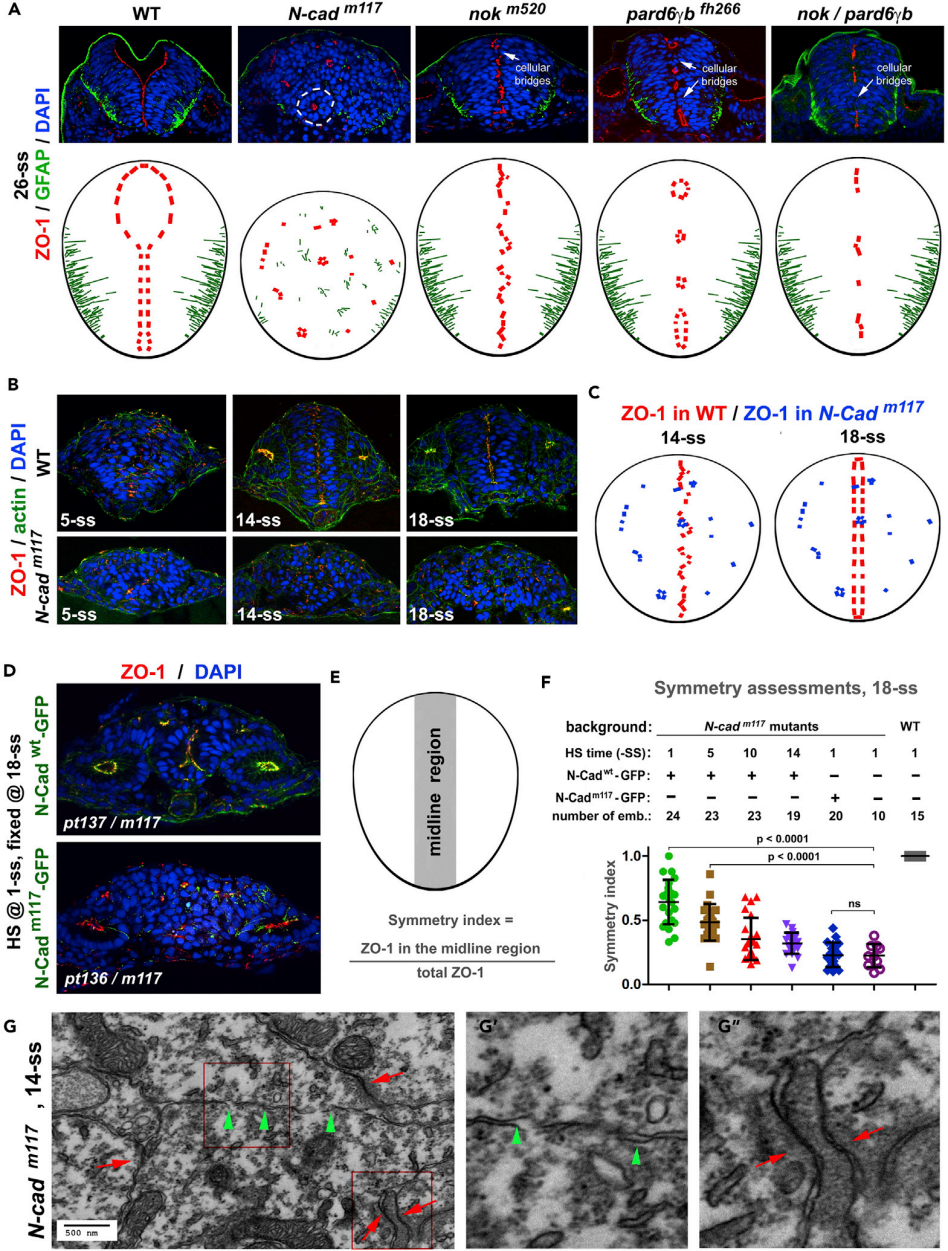
Pioneer Proteins but not Intermediate Proteins Are Required to Establish the Mirror Symmetry of the Neural Tissue (A) In 26-ss *N-Cad*^*m117*^ mutants, apical marker ZO-1 and basal marker GFAP localized ectopically, and cells organized into cellular rosettes (circled by dashed lines), indicating the loss of the mirror symmetry. By contrast, the mirror symmetry remained in 26-ss *nok*^*m520*^, *pard6*^*fh266*^*,* and *nok/pard6γb* mutants, despite the presence of cellular bridges (arrows). Top panels, immunohistochemical images; bottom panels, drawings of the distributions of apical and basal markers. (B) ZO-1 did not align at the midline in *N-Cad*^*m117*^ mutants at 14-ss and 18-ss. (C) Drawings contrast the distributions of apical marker ZO-1 in *N-Cad*^*m117*^ mutants (blue) and wild-type embryos (red) at 14-ss and 18-ss. (D) Apical marker ZO-1 localized more frequently in the midline region in *N-Cad*^*m117*^ mutants that expressed N-Cad^wt^-GFP (in the *Tg(HSP70:N-Cad*^*wt*^*-GFP)*^*pt137*^ transgenic background) than in *N-Cad*^*m117*^ mutants that expressed N-Cad^m117^-GFP (in the *Tg(HSP70:N-Cad*^*m117*^*-GFP)*^*pt136*^ transgenic background). (E and F) To quantify the effects of rescuing expression of N-Cad^wt^-GFP (D) on midline distribution of ZO-1, we devised a symmetry index (E), where the midline region is defined as the midline-striding vertical strip of two nuclear diameters wide. (F) Statistical significance was evaluated by one-way ANOVA and Tukey's post hoc analysis. The individual-value bar graphs represent the symmetry indexes of rescued embryos (with means ± SEM). ANOVA, analysis of variance; SEM, standard error of the mean. (G–G″) In *N-Cad*^*m117*^ mutants at 14-ss, the PAA-like structures (red arrows, G″) were detectable under TEM, but no apparent adhesion structures could be identified on the opposing apical surfaces (green arrowheads, G′). G′ and G″ are magnifications of the boxed regions in (G). Also see Figure S3.

In the *N-Cad*^*m117*^ mutants, no apparent midline ventricles developed at the tissue level at the neural keel, rod, and tube stages, and the neural tissue was 1.5–2 times wider than in the wild-type *nok*^*m520*^ and *pard6γb*^*fh266*^ single mutants and *nok*^*m520*^*/pard6γb*^*fh266*^ double mutants (double mutants were analyzed to assay whether *nok* and *pard6γb* function in the same pathway during neurulation), suggesting that cells could not converge effectively toward the midline (Figures S3A and S3B; Lele et al., 2002, Malicki et al., 2003, Hong and Brewster, 2006). At the cellular and subcellular levels, *N-Cad*^*m117*^ mutant neuroepithelial cells did not align opposingly along the midline. Rather, they aggregated to form numerous rosettes, in which the apical ends of cells pointed to the center to encircle a miniature lumen (Figure 3A, dashed lines; Figure S3C, dashed lines; Lele et al., 2002, Malicki et al., 2003); in addition, the basal marker GFAP often localized ectopically in the interior of the neural tissue (Figure 3A). Thus N-Cad is required for forming the mirror-symmetric neural tube.

Unlike in *N-Cad*^*m117*^ mutants, in 26-ss *nok* and *pard6γb* single mutants as well as in *nok/pard6γb* double mutants, although the lumen failed to emerge, cell convergence toward the midline was not affected (Figures S3A and S3B). Moreover, ZO-1 still localized apically at the midline region and GFAP localized basally, suggesting that the initiation of mirror symmetry does not require Nok and Pard6γb. However, the midline localization of ZO-1 was interrupted by midline-striding cells (Figure 3A; Munson et al., 2008), which we define here, for easy reference, as “cellular bridges”—a midline-striding passage of cell bodies, which is either at least two cell bodies wide and contains no apical markers in the interior of the passage or is single cell body wide but flanked dorsally and ventrally by miniature lumens. The fact that *nok/pard6γb* double mutants did not display more severe midline phenotypes than either *pard6γb* or *nok* single mutants suggests that Nok and Pard6γb work in the same pathway and not in two parallel pathways during neurulation. Despite the midline localization of ZO-1 in these mutants at 26-ss, ZO-1 sites had dispersed away from the midline by 34 hpf, except for *pard6γb* single mutants (Figure S3C). These observations suggest that intermediate proteins are required for the maintenance but not the initiation of mirror symmetry.

To understand when exactly N-Cad is required to initiate the mirror symmetry, we next examined ZO-1 distributions in *N-Cad*^*m117*^ mutants at 5-ss, 14-ss, and 18-ss. We found that unlike in wild-type, ZO-1 remained scattered in the mutants at 14-ss and onward (Figures 3B and 3C), suggesting that the requirement of N-Cad for mirror symmetry is already manifested in the mutants at early neural rod stages. To further confirm this early requirement, we generated stable transgenic fish *Tg(HSP70:N-Cad*^*wt*^*-GFP)*^*pt137*^ and *Tg(HSP70:N-Cad*^*m117*^*-GFP)*^*pt136*^ to examine the effects of expressing N-Cad^wt^-GFP and N-Cad^m117^-GFP at 1, 5, 10, or 14-ss on the symmetry formation in *N-Cad*^*m117*^ mutants. Results showed that inducing N-Cad^wt^-GFP but not N-Cad^m117^-GFP at 1-ss best rescued the mirror symmetry (Figures 3D–3F), suggesting that the initiation of neural rod symmetry requires N-Cad even at the neural keel stages.

It was reported that C-division plays an essential role in the generation of mirror symmetry of the neural rod (Tawk et al., 2007). To determine whether N-Cad-mediated symmetry formation requires C-division, we examined the distribution of apical marker ZO-1 and N-Cad-GFP in both wild-type and *N-Cad*^*m117*^ mutants that were treated with DNA synthesis inhibitors from 1-ss to 18-ss to block cell division. We found that in wild-type, apical markers localized as a jaggy line at the midline region at 14-ss and then segregated into two parallel lines at 18-ss whether treated with DNA synthesis inhibitors or not; by contrast, in *N-Cad*^*m117*^ mutants, apical markers scattered throughout the tissue at both 14-ss and 18-ss regardless of the DNA synthesis inhibitor treatment (Figure S3D). Moreover, in the absence of cell division, the PAAs and OAAs developed normally in wild-type at 14-ss and the OAAs dissolved at 18-ss (magnified panels, Figure S3D). These observations suggest that N-Cad is required for establishing the mirror symmetry of the neural rod regardless of C-division.

These effects of *N-Cad*^*m117*^ mutation on mirror symmetry formation suggest that the strand-swapping *trans*-dimerization of N-Cad on the opposing apical surfaces plays an essential role in mirror symmetry formation because *N-Cad*^*m117*^ mutation causes the Trp2Gly substitution, which blocks such dimerization (Malicki et al., 2003, Tamura et al., 1998, Pertz et al., 1999). Supporting this notion, TEM revealed no apparent electron-dense adhesions at the opposing interfaces between 17 cells that displayed PAA-like structures in two 14-ss *N-Cad*^*m117*^ mutants (Figures 3G–3G″; the 17 cells were identified from 249 TEM micrographs that imaged about a thousand cells.). Thus the PAAs and OAAs adhere cells together opposingly and automatically establish the mirror symmetry of the early neural rod.

### Pioneer Proteins Translocate Apically to Establish the PAAs and OAAs

The OAAs coincide with the axis of this mirror symmetry and are apparently its structural basis. Therefore, both understanding the mechanisms by which pioneer proteins establish the OAAs and determining whether the PAAs facilitate the OAAs go directly to the heart of symmetry genesis. Thus we next examined the dynamics of fluorescent protein-tagged ZO-1 and N-Cad during PAA and OAA formation.

**Figure 4:**
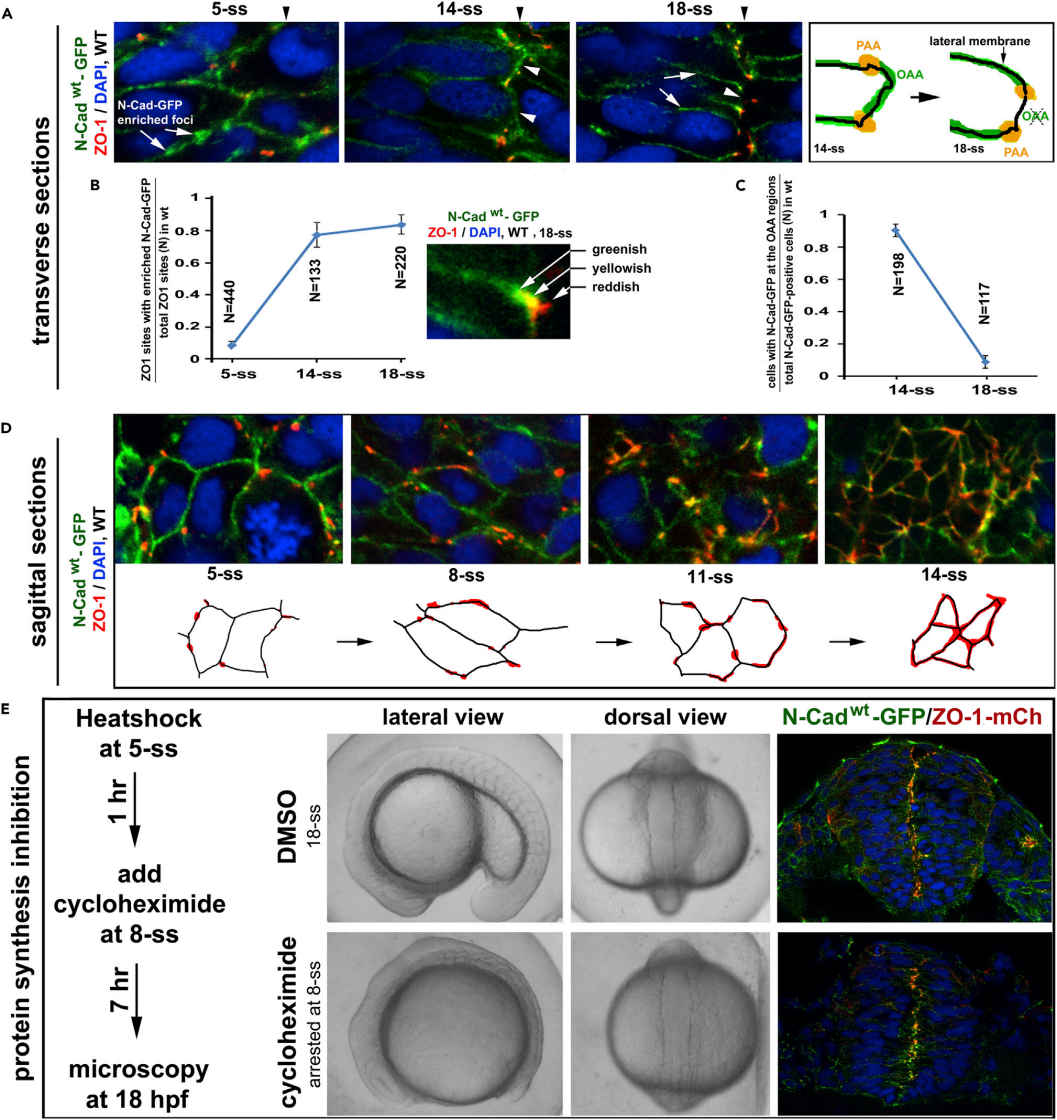
Pioneer Proteins Translocate Apically to Initiate the PAAs and OAAs (A) Dynamic distribution of N-Cad^wt^-GFP in *Tg(HSP70:N-Cad*^*wt*^*-GFP)*^*pt137*^: at 5-ss, ubiquitously on the cell membrane, with numerous N-Cad^wt^-GFP-enriched foci; at 14-ss, enriched at the PAAs and on opposing apical surfaces (arrowheads); at 18-ss, enriched at the PAAs and weakly present on the lateral membranes (white arrows), but absent on the opposing apical surfaces (arrowheads; drawings on the right). Black arrowheads indicate the positions of the midline axis. (B) A line graph displays the increase in the ratios between ZO-1 sites that were tightly associated with N-Cad^wt^-GFP-enriched foci and total ZO-1 sites from 5-ss to 18-ss (8 embryos for each stage; means ± SEM). The inset illustrates the tight association, but not 100% co-localization, between ZO-1 and N-Cad^wt^-GFP at the PAAs, resulting in greenish, yellowish, and reddish signal appearances. SEM, standard error of the mean. (C) A line graph displays the reduction in the ratios between cells with N-Cad^wt^-GFP on the opposing apical surfaces and total N-Cad^wt^-GFP-positive cells from 14-ss to 18-ss (8 embryos for each stage; mean ± SEM). (D) Sagittal imaging revealed that during neural keel-early neural rod transition, ZO-1 signals changed from small punctate sites to circumferential belts (illustrated by drawings). (E) The development of *Tg(HSP70:N-Cad*^*wt*^*-GFP)*^*pt137*^/*Tg(HSP70: ZO-1.1-mCherry)*^*pt117b*^ double transgenic embryos was arrested at 8-ss (30 embryos) when treated with protein synthesis inhibitor cycloheximide, but the development proceeded normally to 18-ss (10 embryos) in the 4% DMSO control condition. The scheme illustrates the treatment procedure. In the presence of cycloheximide, N-Cad^wt^-GFP and ZO-1-mCherry (ZO-1-mCh) still localized to the midline region (compare with N-Cad-GFP and ZO-1 distributions in untreated embryos at 8-ss and 18-ss, Figure S4B). Also see Figure S4.

N-Cad^wt^-GFP, expressed in *Tg(HSP70:N-Cad*^*wt*^*-GFP)*^*pt137*^, localized ubiquitously on the cell membranes at 5-ss and gradually enriched at both the PAAs (ZO-1 positive) and the opposing apical surfaces by 14-ss; then at 18-ss, N-Cad^wt^-GFP diminished at the opposing apical surfaces but remained enriched at the PAAs and was weakly present on the lateral membranes (Figures 4A–4C and S4A–S4C). These dynamics corroborated the N-Cad distribution patterns revealed by immunohistochemistry (Figures 1A and S1A).

While N-Cad^wt^-GFP became enriched at the apical region, ZO-1 punctate sites also became enriched at the midline region; this midline enrichment of ZO-1 and N-Cad^wt^-GFP occurred earlier in the ventral region than in the dorsal regions (Figure S4B). The ZO-1 punctate sites were smaller at earlier stages than at later stages and became more associated with N-Cad^wt^-GFP over time (Figures 4B and S4C); eventually, these ZO-1 sites fused into circumferential belts at the apical ends, marking the formation of the primitive PAAs, which segregate the apical cell membranes from the lateral cell membranes (Figure 4D; Videos S1, S2, and S3). The formation of the primitive PAAs also mark the segregation of membrane-bound N-Cad into two domains: some N-Cad molecules localize apical to the ZO-1-positive TJs of the primitive PAAs, whereas other N-Cad molecules localize basal to the ZO-1 positive TJ of the primitive PAAs. N-Cad localizing apical to ZO-1 participates in the formation of the OAAs (Figure 4A, 14-ss), and N-Cad localizing basal to ZO-1 participates in the formation of the AJs of the primitive PAAs. Thus this segregation of N-Cad into two groups lays down the ground for their differential regulation.

At 14-ss, the PAAs first aligned in a jaggy line at the midline region, making it difficult to distinguish the left PAAs from the right ones under confocal microscopy (this very jaggy PAA alignment suggests that left and right tissue halves are adhered together at the opposing apical surface, just as it is difficult to draw a straight line to separate a group of soap bubbles that are stuck together into left and right halves); however, at 18-ss, the left and right PAAs segregated from each other into two midline-flanking planes, giving the appearance of smooth alignment, which suggests that left and right halves have separated from each other, and the surface tension makes the apical surface smooth and the PAAs flank the midline in two parallel lines (Figures 4A and 3C).

To visualize that ZO-1 and N-Cad enrich apically in action, we followed N-Cad^wt^-GFP and ZO-1-mCherry in live *Tg(HSP70:N-Cad*^*wt*^*-GFP)*^*pt137*^/*Tg(HSP70: ZO-1.1-mCherry)*^*pt117b*^ double transgenic embryos. We found that the foci of both proteins generally moved in the basal-to-apical direction—although they often traveled basally briefly before moving apically again—and fused into the PAAs and OAAs by 14-ss; in addition, their apical enrichments occurred earlier in the anterior regions than in the more posterior regions (Videos S4, S5, and S6). Finally, the left and right PAAs were separated into two planes at the late neural rod stages (Video S7). To determine whether apical enrichment of N-Cad and ZO-1 requires new protein synthesis, we next treated the embryos with protein synthesis inhibitor cycloheximide; we found that both N-Cad-GFP and ZO-1-mCherry still enriched apically (Figure 4E), suggesting that N-Cad and ZO-1 could enrich at the midline region by apical translocation to initiate the PAAs and OAAs; accompanying the formation of the PAAs and OAAs was the establishment of the mirror symmetry, which was also manifested by equal distribution of cells on the two sides of the midline (Figure S4D).

### Dissolution of the OAAs

**Figure 5:**
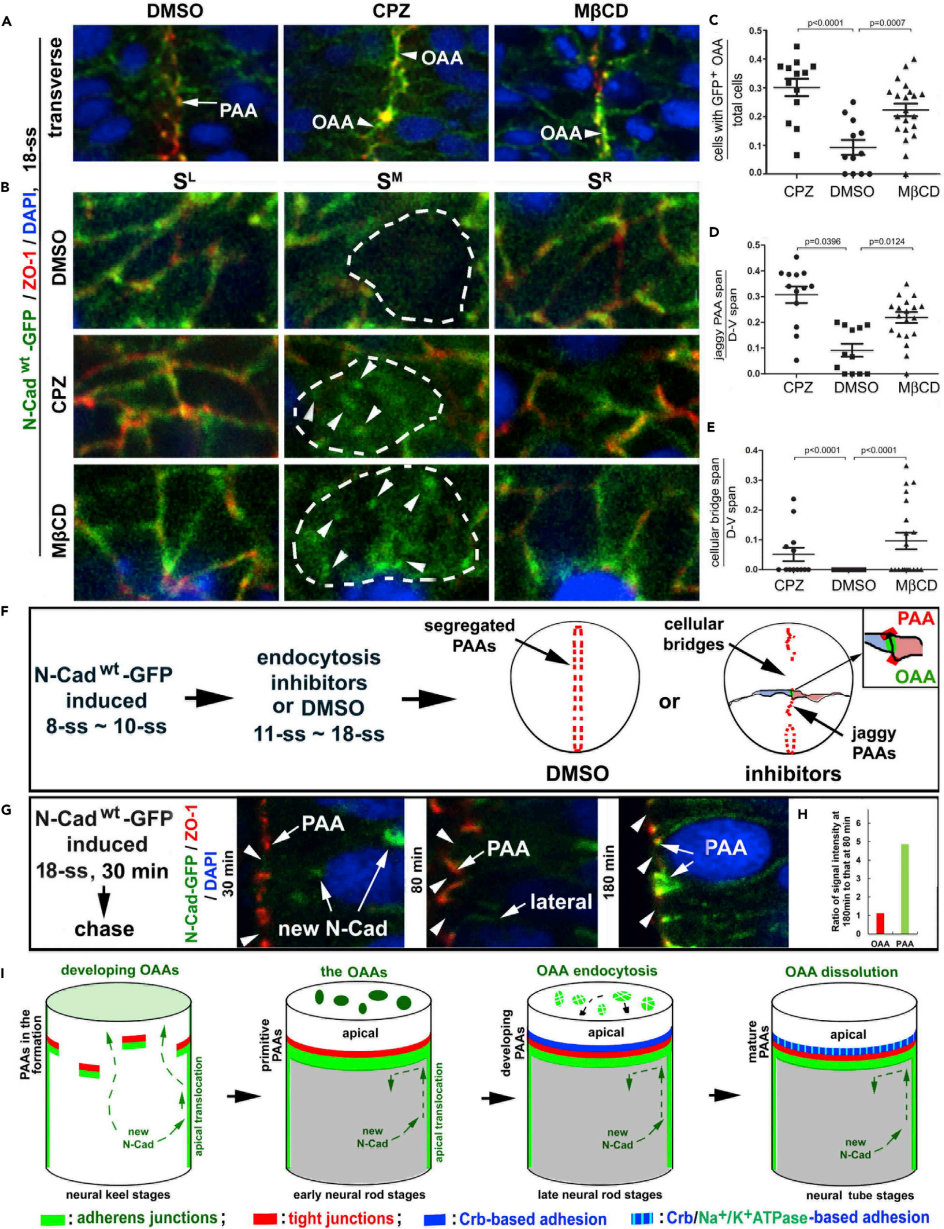
OAA Dissolution Depends on Endocytosis and Restriction of Apical Translocation of N-Cad (A) Effects of treatments with endocytosis inhibitors CPZ (N = 13 embryos) and MβCD (N = 20 embryos) on the apical adhesions in *Tg(HSP70:N-Cad*^*wt*^*-GFP)*^*pt137*^ embryos at 18-ss. Note that inhibition of endocytosis resulted in jaggy midline alignment of ZO-1 and accumulation of N-Cad-GFP at the opposing apical surface (arrowheads); however, in DMSO controls, the PAAs (arrows) segregated into two parallel planes flanking the midline and the OAAs dissolved. Panels are magnifications of the boxed regions in the bottom row of panels in Figure S5F. (B) Serial sagittal imaging of the apical adhesions revealed the retention of the N-Cad-GFP at the OAAs (arrowheads, S^M^ sections) in embryos treated with either CPZ (N = 12 embryos) or MβCD (N = 12 embryos), but not in DMSO control (N = 4 embryos). The opposing apical surfaces are circled with dashed lines. (C–E) Individual-value bar graphs (with means ± SEM) illustrate the ratios between cells displaying N-Cad^wt^-GFP signals at the OAA region and total cells (C), the ratios between jaggy ZO-1 positive PAA span and dorsal-ventral (D-V) span (D), and the ratios between cellular bridge span and D-V span (E). The numbers of Type I and II embryos analyzed (Figures S5B and S5C) are 13 for CPZ, 20 for MβCD, and 12 for DMSO. *p* Values by two-tailed Student's t test. (F) A scheme summarizes the treatments and effects described in A–D. (G and H) N-Cad^wt^-GFP, when heat-shock-expressed for 30 min (starting at 18-ss), first appeared inside the cells 30 min later (arrows new N-Cad), then on the lateral cell membranes 80 min later (arrow lateral), and finally enriched at the PAAs (arrows PAA) 180 min later. Note that N-Cad^wt^-GFP never localized to the opposing apical surfaces (arrowheads) at any time. The changes of N-Cad^wt^-GFP signal intensities at the apical regions were quantified as ratios between 180-min chase (N of OAA sites = 59; N of PAA sites = 118) and 80-min chase (N of OAA sites = 45; N of PAA sites = 90); note no changes at the opposing apical surfaces and a five times increase at the PAAs (H). (I) Diagrams summarize the formation of primitive, developing, and mature PAAs, which block the apical translocation of newly synthesized N-Cad from the lateral membranes; the OAAs eventually dissolve by endocytosis. Also see Figure S5.

After the neuroepithelial cells reorganize into the early neural rod, the OAAs need to dissolve to allow subsequent lumen formation. OAA dissolution is likely mediated by endocytosis but not ectodomain shedding because no GFP signals were present at the opposing apical surfaces by 18-ss (Figure 4A) and because various cadherins can be removed from cell membrane by endocytosis in other cellular contexts (Kowalczyk and Nanes, 2012, Cadwell et al., 2016, Le et al., 1999). To test this hypothesis, we next blocked clathrin-dependent endocytosis with chlorpromazine hydrochloride (CPZ) and clathrin-independent caveolar endocytosis and macropinocytosis-like pathways with methyl-β-cyclodextrin (MβCD) (Dutta and Donaldson, 2012). We found that both CPZ and MβCD treatments distorted the central nervous system and hindered neural tube lumen formation in about 50% of the embryos (Types I, II, and III) (Figures S5A–S5C). Transverse examination of apical adhesion morphologies of Type I and II treated embryos at 18-ss under confocal microscopy revealed that the OAAs existed in extended areas and that the ZO-1-based left and right PAAs often failed to segregate as in dimethyl sulfoxide (DMSO) controls; instead, they displayed a jaggy alignment (Figures 5A, 5C, 5D, and S5F), which normally only exists in wild-type at the early neural rod stage. Sagittal imaging also revealed apparent OAAs at the midline plane (Figure 5B). These data suggest that inhibition of endocytosis delayed OAA dissolution (Figure 5F). Moreover, CPZ and MβCD treatments introduced cellular bridges at 18-ss (Figures S5F, 5E, and 5F). These effects of endocytosis inhibitors on OAA dissolution are unlikely due to secondary effects caused by drug inhibition of cell division because both the numbers of M-phase nuclei and total nuclei per tissue section were not affected by CPZ and MβCD treatments (Figures S5D and S5E).

Besides endocytotic removal of N-Cad at the OAA regions, preventing newly synthesized N-Cad from translocating from the lateral membranes to the apical membranes would also contribute to OAA dissolution. First, we needed to determine whether newly synthesized N-Cad is targeting to the lateral membranes but not to the opposing apical surfaces, as newly synthesized E-cadherin is in MDCK cells (Chen et al., 1999). Thus we induced N-Cad^wt^-GFP transiently at 18-ss and then mapped its fate. Indeed, N-Cad^wt^-GFP localized first on the lateral membrane and then enriched at the PAAs, but never localized on the opposing apical surfaces (Figures 5G–5I, and S5G), suggesting that newly synthesized N-Cad did not accumulate in the opposing apical membranes and that the PAAs may prevent newly synthesized N-Cad from translocating from the basolateral membranes to the opposing apical surfaces. However, when induced before the PAAs were established, N-Cad^wt^-GFP could localize to the OAA regions at the early neural rod stage (Figure 4A).

Together, these observations suggest that sustained OAA dissolution is the outcome of the combination of four events: endocytotic removal of N-Cad from the opposing apical membranes (it is unclear whether internalized N-Cad is recycled for constructing the PAAs or is simply destroyed), the initial targeting of newly synthesized N-Cad to the basolateral membranes, translocation of N-Cad from the lateral membranes to the PAAs, and the likely blockade of N-Cad^wt^-GFP's translocation to the opposing apical surfaces by developing and mature PAAs (Figure 5I).

### The Roles of Intermediate Proteins in Maintaining the PAAs

At the level of subcellular adhesions, whereas the OAAs dissolve during the transition from the early neural rod to the late neural rod, the PAAs persist. It is during this transition when intermediate proteins localize to the apical regions. Thus do intermediate proteins maintain the PAAs?

**Figure 6:**
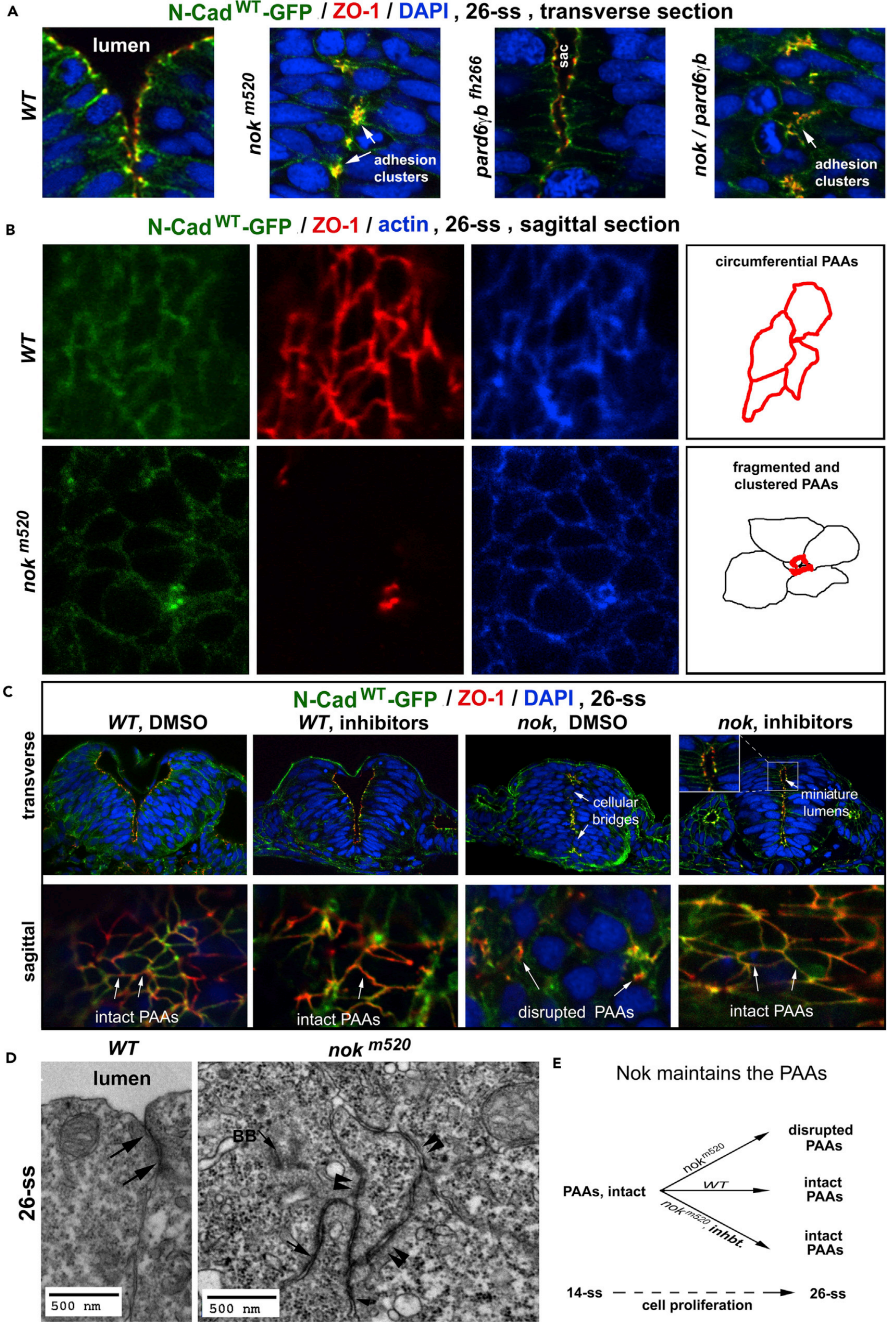
Intermediate Proteins Nok and Pard6 Stabilize the PAAs (A) Transverse imaging: In 26-ss *nok*^*m520*^ single and *nok*^*m520*^/*pard6γb*^*fh266*^ double mutants, ZO-1 and N-Cad^wt^-GFP aggregated in clusters (arrows) at the midline region, compared with the regular distribution of the PAAs in 26-ss wild-type. In 26-ss *pard6γb*^*fh266*^ mutants, the PAAs contoured small luminal sacs. (B) Sagittal imaging: As summarized by the drawings on the right, in 26-ss wild-type embryos, ZO-1 and N-Cad^wt^-GFP enriched at the PAAs as circumferential belts; by contrast, in 26-ss *nok*^*m520*^ mutants, ZO-1 and N-Cad^wt^-GFP clustered at the corners of the cells, indicating the loss of PAA integrity. (C) ZO-1 and N-Cad^wt^-GFP distributions (top panels, transverse imaging; bottom panels, sagittal imaging) in DMSO-treated and DNA-synthesis-inhibitor-treated wild-type and *nok*^*m520*^ mutants (6 wild-type and 12 mutant embryos for each condition). Note that inhibitor treatment of *nok*^*m520*^ mutants restored intact PAAs and miniature lumens (inset, arrow miniature lumens) and prevented cellular bridges (arrows cellular bridges). (D) TEM: Unlike in wild-type, where the PAAs sealed the paracellular clefts near the lumen (arrows), cell-cell junctional complexes clustered at membrane protrusions in *nok*^*m520*^ and indiscriminately adhered cells from both sides together at 26-ss (double arrowheads). Arrow BB, basal body. (E) A scheme depicts that Nok is required to maintain the PAAs during cell proliferation. Inhbt., DNA synthesis inhibitors. Also see Figure S6.

To answer this question, we next examined more closely the effects of the loss of Nok and Pard6γb on the PAAs. In *nok* mutants, the normal circumferential PAAs were lost and residual adhesions clustered to a corner of neighboring cells (Figures 6A, 6B, and S6A). By contrast, in *pard6γb*^*fh266*^ single mutants, the circumferential PAAs of individual cells were largely intact and miniature lumens developed, despite the disruption of the overall integrity of the PAAs by cellular bridges, which suggest that Pard6γb is required for keeping the PAAs of individual cells as an intact network in the left and right halves of the tissue (Figure 6A). However, in *pard6γb*^*fh266*^/*nok* double mutants, the PAA integrity was disrupted as severely as in *nok* single mutants but more than in *pard6γb*^*fh266*^ single mutants, suggesting again that Nok and Pard6γb function in the same genetic pathway. It is worth noting that the miniature lumen phenotype in *pard6γb*^*fh266*^ single mutants did not appear to develop in *pard6γb*^*s441*^ mutants even at 24 hpf (2 hr after 26-ss; Munson et al., 2008); the lesser degree of disruption of the PAAs in *pard6γb*^*fh266*^ than in *pard6γb*^*s441*^ suggests that *pard6γb*^*fh266*^ is a hypomorphic mutation. Because intact PAAs have already been established by the time intermediate proteins localize to the apical regions (Figure 1G), the above observations suggest that both Nok and Pard6γb are collectively required to maintain the integrity of the PAAs.

Why is Nok required to maintain the PAAs, which are initially able to form without it (Figures S6B and S6C)? During neurulation, the integrity of the PAAs is constantly challenged by cell divisions because the circumference of the PAAs periodically increases and decreases depending on the locations of the cell nuclei during the cell cycle and because new PAAs need to be established in newly generated cells (Chenn et al., 1998). Perhaps Nok helps with coping with such disturbances. To test this idea, we next blocked cell division in *nok* mutants with DNA synthesis inhibitors aphidicolin and hydroxyurea from 10-ss to 26-ss and examined the effects on PAA integrity at 26-ss. Unlike in DMSO-treated control *nok* mutants, inhibition of cell division rescued the PAA integrity defect in *nok* mutants (Figures 6B and 6C), suggesting that Nok is required to cope with the disturbance of PAA integrity by cell division. In addition, the cellular bridge defects were eradicated, and the lumen formed, although the lumen is smaller than that in wild-type (Figures 6B and 6C), suggesting that the dissolution of the OAAs also proceeded normally in the absence of cell division in *nok* mutants.

We next examined the loss-of-Nok effects on the cell-cell adhesions under TEM. We found clusters of electron-dense cell-cell adhesion complexes adhering membrane protrusions of neighboring cells in a plywood-like configuration; these adhesion clusters resembled the ZO-1- and N-Cad-positive clusters under immunohistochemistry. More importantly, these adhesion clusters indiscriminately adhered cells of both the same and opposing orientations (Figures 6D, S6D, and S6E).

Thus these data suggest for the first time that intermediate protein Nok maintains the PAAs by enhancing their resilience to the disturbance caused by cell divisions, ensuring the tissue integrity *within* the left and right halves of the tissue as well as the separability *between* the left and right halves (Figure 6E).

### Terminal Proteins Inflate the Neural Tube Lumen by Generating Osmotic Pressure

**Figure 7:**
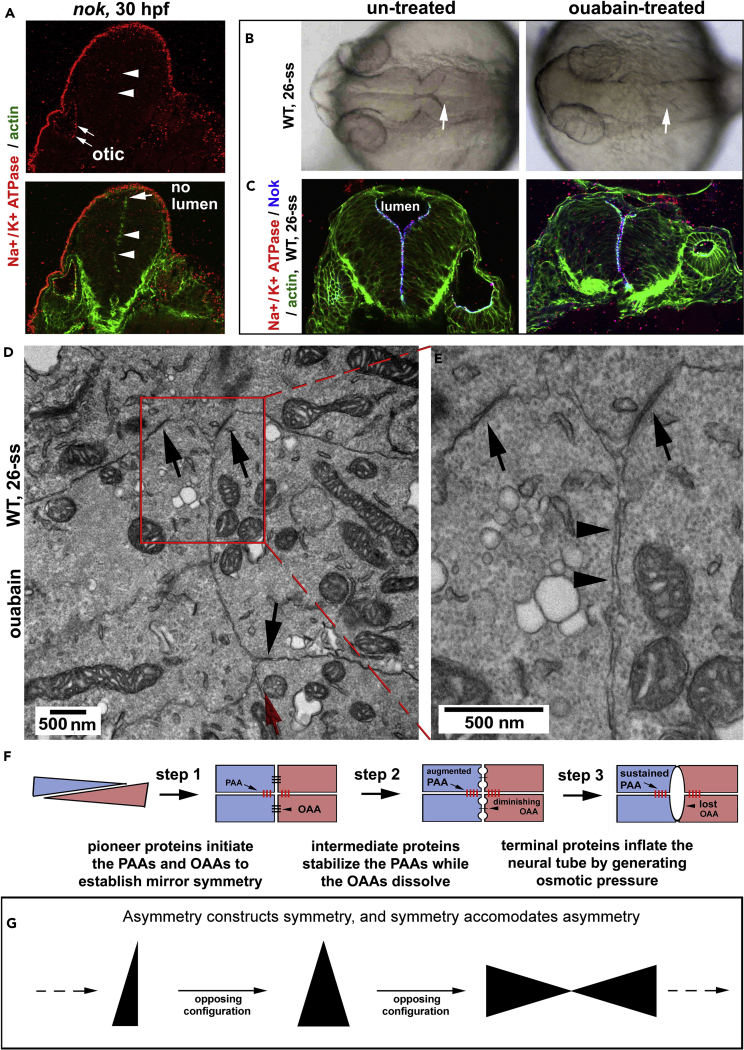
Terminal Protein Na^+^/K^+^-ATPase Is Required for Luminal Inflation but Not for OAA Dissolution (A) In 30-hpf *nok*^*m520*^ mutants, Na^+^/K^+^-ATPase α failed to enrich at the midline region in the neural tissue (arrowheads) but still enriched in the otic vesicle (arrows). (B) Brain ventricle inflation was blocked by ouabain treatment (arrows; 26-ss). (C) Ouabain did not prevent Na^+^/K^+^-ATPase α from enriching apically with Nok in the neural tissue (26-ss). (D and E) TEM revealed that in ouabain-treated embryos, the PAAs persisted (black arrows), whereas the OAAs were not detectable on the opposing apical cell membranes (arrowheads). (E) Magnification of the boxed region in (D). The red arrow indicates the midline direction. (F) Diagrams summarize that pioneer, intermediate, and terminal proteins regulate zebrafish neurulation through the three steps. (G) A diagram illustrates the three core elements that underlie symmetry formation: symmetry, asymmetry, and opposing configuration. Also see Figure S7.

Among all the polarity proteins examined in this study, terminal protein Na^+^/K^+^-ATPase α is the last to enrich apically (Figures 1B, 1G, S1B, and S1C). This late onset makes sense because Na^+^/K^+^-ATPase is required to generate extracellular osmotic pressure to inflate the lumen and because this apical luminal pressure depends on a TJ-based paracellular fluid barrier (Lowery and Sive, 2005, Lowery and Sive, 2009, Bagnat et al., 2007, Krupinski and Beitel, 2009, Chang et al., 2012), whose very structural integrity and impermeability also require Na^+^/K^+^-ATPase, possibly through its β subunit's homophilic *trans*-adhesion capability (Cereijido et al., 2012, Vagin et al., 2012). Here we show that the late onset of Na^+^/K^+^-ATPase's apical enrichment is at least partially ensured by its requirement for Nok and Pard6γb because the loss of Nok and Pard6γb blocked or delayed Na^+^/K^+^-ATPase α from enriching apically in the neural tissue (Figures 7A, 7C, and S7A–S7D); by contrast, Na^+^/K^+^-ATPase α could still localize apically in cellular rosettes in *N-Cad*^*m117*^ mutants (Figures S7A–S7E), where Nok is also enriched (Zou et al., 2008). It is worth pointing out that Na^+^/K^+^-ATPase still localized to the apical surface in the otic vesicle in *nok* mutants (Figure 7A), suggesting that Nok's targeting function was not required or substituted by another unknown protein in the otic vesicle. The impediment of Na^+^/K^+^-ATPase α′s apical enrichment in *nok* and *pard6* mutants was unlikely due to the absence of the protein because Na^+^/K^+^-ATPase was expressed normally in these mutations (Figure S7A).

The late onset of Na^+^/K^+^-ATPase's apical localization also raised the question of whether luminal pressure contributes to OAA dissolution by forcing them apart. To answer this question, we next treated embryos with ouabain to block the ion-pumping function of Na^+^/K^+^-ATPase (Sandtner et al., 2011) and then examined apical adhesions at 26-ss. We found that ouabain severely hindered lumen inflation at 26-ss as expected (Figure 7B; Lowery and Sive, 2005), even though Na^+^/K^+^-ATPase α properly localized to the apical surface and co-localized with Nok, as in untreated embryos (Figures 7C, S7B, and S7F). This lumen inflation defect was not simply due to developmental delay because it persisted even at 34 hpf (Figures S7G and S7H). Despite this luminal defect, the OAAs dissolve normally because unlike the PAAs, no electron-dense OAAs were detectable under TEM, even though the opposing apical cell membranes remained juxtaposed (Figures 7D and 7E, a total of 37 opposingly posed cells from two embryos were examined under TEM; compare with the wild-type, in which an average of 1–2 OAAs can be found on the opposing apical surface of each cell, Figures 1E and 1E′). These results suggest that OAA dissolution does not require Na^+^/K^+^-ATPase-mediated luminal fluidal pressure, which simply inflates the luminal space to transform the smooth neural rod into the neural tube (Figure 7F).

## Discussion

In this study, we tested the hypothesis that during zebrafish neurulation, apical polarity proteins localize in an orderly manner to the apical regions of neuroepithelial cells to orchestrate the genesis of the mirror-symmetric neural rod and neural tube by dynamically regulating apical cell-cell adhesions. Using genetic, transgenic, and imaging approaches, we revealed that these apical polarity proteins localize in a three-step spatiotemporal order: pioneer, intermediate, and terminal. By these steps (Figure 1), pioneer proteins first initiate the PAAs and the OAAs, which cohere asymmetric cells into the mirror-symmetric neural rod (Figure 2, Figure 3, Figure 4); subsequently, intermediate proteins augment the PAAs while the OAAs dissolve (Figures 5 and 6); finally, terminal proteins are required to inflate the lumen by generating extracellular osmotic pressure (Figure 7). By this framework, asymmetric neuroepithelial cells organize into the mirror-symmetric neural rod and then into the neural tube.

### The Rigor and Limitations of the Three-Step Localization Order of Apical Polarity Proteins

The spatiotemporal order of apical polarity protein localization concerns the four cornerstones of tissue morphogenesis—space, time, structure, and reconstruction; thus it is vital to characterize this order as rigorously as possible. To do so, we applied three precautions: (1) to ensure that the same neural tissue region was examined, we used the adjacent otic vesicle as a landmark; (2) to relieve the interferences from developmental variations among embryos, we visualized two or more proteins simultaneously in the same embryos to reveal the relative order of their localizations; and (3) to reveal proteins' subcellular localization, we relied mainly on immunohistochemistry of endogenous proteins and used transgenic fluorescent fusion proteins as auxiliary and confirming measures. With these precautions, we revealed that pioneer proteins (N-Cad, E-Cad, β-catenin, F-actin bundles, and ZO-1), intermediate proteins (Crb proteins, Nok, Lin7c, Pard3, Pard6γb, and aPKC), and terminal proteins (Na^+^/K^+^-ATPase) sequentially enrich or localize to the apical regions to regulate apical cell-cell adhesions for proper neurulation (Figures 1 and S1). Although these proteins only represent a fraction of all apical polarity proteins, our study has established a framework by which to understand the localization dynamics of other apical polarity proteins. It is worth pointing out that within each of the three categories, protein localization may follow certain hierarchical suborders. In addition, it would also be interesting to understand how the stepwise localization of apical polarity proteins is coordinated with the localization of basolateral polarity proteins and planar polarity proteins.

### Step 1: Pioneer Apical Polarity Proteins Initiate the PAAs and OAAs to Establish the Mirror Symmetry of the Neural Rod

During the neural keel-neural rod transformation, cell-cell adhesions need to be modulated so that cells can reposition themselves to take on a new cytoarchitecture. This modulation is achieved by translocating pioneer proteins from the lateral cell membranes to the apical cell membranes to form the primitive PAAs (including the AJs and TJs) and OAAs (AJ-like), thus shifting the cell-cell adhesion balance toward the apical ends (Figures 1, 2, 4, and S4). The apical enrichment of the pioneer proteins, hence the formation of the primitive PAAs and OAAs, spreads in the ventral-to-dorsal and anterior-to-posterior directions (Figure S4; Videos S4 and S5). The resulting PAAs and OAAs function differently: the PAAs hold together cells *within* the left and right halves of the tissue; by contrast, the OAAs stitch together cells *between* the left and right halves of the tissue. Thus, like closing zippers, the primitive PAAs and OAAs stick together cells of opposing orientations at the midline, transforming the neural keel into the mirror-symmetric early neural rod, with the OAAs constituting the axis of the mirror symmetry.

### Step 2: Intermediate Apical Polarity Proteins Stabilize the PAAs while the OAAs Dissolve

During the transition from early neural rod to late neural rod, intermediate proteins directly augment the PAAs but indirectly facilitate OAA dissolution. Intermediate protein Nok may augment the PAAs by recruiting the cell-cell adhesion molecules Crb1 and Crb2a to the PAAs (Figures 2E and S2E; Zou et al., 2012, Zou et al., 2013). Furthermore, Pard6γb may strengthen the PAAs by physically interacting with Nok, as in MDCK cells where Pard6γb homolog Par6 directly interacts with Nok homolog PALS1 to control TJ assembly (Hurd et al., 2003). Loss of intermediate proteins, particularly Nok, causes the PAAs to fragment and indiscriminately adhere cells of both parallel and opposite orientations, making the left and right halves of the tissue inseparable (Figures 6A, 6B, and 6D). Although *nok*^*m520*^ mutation causes more severe defects than *pard6γb*^*fh266*^ mutation, possibly due to the hypomorphy of *pard6γb*^*fh266*^ mutation or *pard6* gene redundancy (Figure 6A; Munson et al., 2008), it is most likely that Nok, Pard6γb, and their partners augment the PAAs collectively in the same pathway.

Intermediate proteins, however, may not directly regulate OAA dissolution because in *nok* mutants, the OAAs develop normally at 14-ss and dissolve properly by 26-ss when cell division was blocked (Figures 6 and S6). Instead, intermediate proteins may indirectly regulate endocytosis-mediated OAA dissolution (Figure 5) by augmenting the PAAs to fully block the supply of N-Cad to the OAA regions from the lateral cell membranes, where the newly synthesized N-Cad first localizes (Figures 5G–5I).

### Step 3: Terminal Protein Na^+^/K^+^-ATPase Inflates the Neural Tube

After OAA dissolution and PAA augmentation by intermediate proteins, terminal protein Na^+^/K^+^-ATPase enriches at the apical surface to mediate ion accumulation in the apical extracellular space, which eventually leads to luminal inflation through osmotic pressure (Figures 7 and S7A–S7F; Lowery and Sive, 2009). The maintenance of osmotic pressure depends on a tight paracellular seal, to which Na^+^/K^+^-ATPase contributes directly (Cereijido et al., 2012, Vagin et al., 2012), leading to the mature PAAs. Thus it is logical for Na^+^/K^+^-ATPase to enrich apically at the end of neurulation, echoing the late enrichment of Na^+^/K^+^-ATPase to the septate junctions in fly epithelia to maintain the paracellular seal (Genova and Fehon, 2003, Paul et al., 2003, Laprise et al., 2009). We found that the late apical enrichment of Na^+^/K^+^-ATPase depends on the intermediate proteins Nok and Pard6γb (Figures 7A and S7B–S7D); this finding differs from Lowery et al.'s conclusion that Na^+^/K^+^-ATPase α′s apical localization does not require Nok in *nok*^wi83^ (Lowery and Sive, 2005), a viral intronic insertional *nok* mutant allele (Wiellette et al., 2004), which still expresses a small amount of Nok (Lowery and Sive, 2005). The requirement of Nok and Pard6γb for the apical enrichment of Na^+^/K^+^-ATPase echoes the indirect interactions between Crb and Na^+^/K^+^-ATPase observed in the fly: Crb recruits FERM domain protein Yurt to the apical membrane at late stages of epithelial differentiation (Laprise et al., 2006); in turn, Yurt physically binds to neuroglian, a partner of Na^+^/K^+^-ATPase (Genova and Fehon, 2003), and vertebrate homology Yurt homolog EPB41L5 is required for the localization of Na^+^/K^+^-ATPase to the lateral membrane in the MDCK cells (Laprise et al., 2009). Supporting the notion that Na^+^/K^+^-ATPase needs Nok to enrich apically, the lumen failed to inflate in *nok* mutants when treated with DNA synthesis inhibitors, even though the integrity of the PAAs was restored (Figure 6C). Conversely, apical localization of Nok and actin does not require Na^+^/K^+^-ATPase (Lowery and Sive, 2005), agreeing with the logic that an earlier event is independent of a later event. Thus our finding that Na^+^/K^+^-ATPase depends on Nok and Pard6γb to enrich apically may suggest for the first time that late onset of Na^+^/K^+^-ATPase's apical enrichment is mediated by similar physical interactions observed in the fly; therefore the processes of physical interactions define an inherent temporal order.

### Coordinating Apical Adhesions with Cell Division in Symmetry Formation during Neurulation

Tissue morphogenesis requires not only cell re-configuration but also a critical mass of cells. Thus it is important to understand how cell divisions are coordinated spatiotemporally with apical adhesions during neurulation. This coordination underlies the delicate balance between tissue plasticity and tissue cohesiveness: a tissue needs to be plastic to allow cells to re-position, to change shapes, and to accommodate new cells so that the tissue can take a new architecture; to maintain integrity, the tissue also needs to be cohesive to cope with the disturbance introduced by cell division, cell re-configuration, and cell shape changes.

During neural keel-early neural rod transition when cells are still in the process of massive relocation and shape changes, the primitive PAAs and OAAs may be strong enough to generate sufficient tissue cohesiveness and may also be plastic enough to maintain the necessary tissue plasticity. During this time, cell division may take C-division as a default mode as a consequence of these adhesions. After a mirror-symmetric neural rod is formed, the tissue needs both to relieve the disturbance introduced by cell division and to further stabilize the newly formed rod architecture. To do so, intermediate proteins start to localize apically to augment and transform the primitive PAAs into the developing PAAs. In conjunction with the dissolution of the OAAs, which establishes an impassible gap between the left and right tissue halves, the augmented developing PAAs may have become strong enough to retain both sister daughter cells on the same side where their mother cell once resides, thus making cell division take the P-division mode. Consistent with this theory, intermediate protein Pard3 is required during this time to rotate the mitotic spindle axis by 90° to undergo P-division (Geldmacher-Voss et al., 2003).

C-division has been proposed by several studies to play an essential role in generating mirror symmetry during neurulation (Tawk et al., 2007, Buckley et al., 2013, Buckley and Clarke, 2014). However, with this theory it is difficult to explain why the mirror symmetry still formed when cell division was inhibited by DNA synthesis inhibitors (Figure S3D; Tawk et al., 2007, Zigman et al., 2011; note that incomplete separation between the left and right tissue halves was observed when cell inhibition was blocked by injecting anti-*emi1* morpholino, which might elicit additional cellular effects besides cell-cycle arrest, Buckley et al., 2013, Wang and Kirschner, 2013) In addition, although C-division produces two daughter cells striding the midline, it is hard to conclude that C-division is sufficient for generating symmetry because it is impossible to separate C-division from other properties of cells, such as N-Cad-mediated apicobasal polarization, which positions dividing cells close to the midline in the first place (Zigman et al., 2011). In addition, non-cell-autonomous regulations need also be considered: a dividing cell is embedded in non-dividing cells, which would send polarity cues to the dividing cells through cell-cell adhesions. Thus in such a complex situation, it would also be a stretch to claim that C-division is sufficient to determine mirror symmetry. Not only is cell division not required for generating mirror symmetry but also it appears to impose a destructive effect on neurulation because PAA integrity defects in both *nok* and *scribble* mutants were rescued when cell division was inhibited (Figure 6C; Zigman et al., 2011). Then what role does cell division play in symmetry formation during neurulation?

Here, we provide an alternative thinking about this question: cell division is neither essential nor necessarily sufficient for symmetry generation; rather, cell division, an essential process to increase tissue size, simply concurs and intermingles with the process of symmetry generation. Cell division may not play a constructive role in this process; rather, it imposes instability that must be stabilized by additional apical adhesions mediated by intermediate polarity proteins. The underlying argument for this thinking is that C-division is not required for the formation of the primitive PAAs and OAAs (Figure S3D), which are the essential structures that polarize cells into asymmetric individuals and, in the meantime, configure them into a mirror-symmetric collective.

Our finding that the apical localization of intermediate proteins, including Pard3, does not concur with C-division differs from the observations made with Pard3-GFP (Tawk et al., 2007, Hong et al., 2010; Buckley et al., 2013). By using Pard3-GFP, expressed via blastomere mRNA injection, Tawk et al. detected Pard3-GFP at the cleavage furrow during C-division, and they suggested that Pard3 plays roles in C-division and thus in mirror symmetry formation (Tawk et al., 2007). However, with no immunohistochemical data to verify that the endogenous Pard3 is present in cells that go through C-division (although their immunohistochemistry showed endogenous Pard3 was absent at neural plate stage and localized apically at late neural rod stage, at 18-ss), such a claim must be interpreted cautiously because mRNA injection results in precocious expression of Pard3-GFP. When precociously expressed, Pard3-GFP has to localize somewhere; it may first localize diffusely and then localize prematurely to ZO-1-positive foci and to the primitive PAAs, generating an illusion that Pard3-GFP plays roles in C-division (Tawk et al., 2007, Chenn et al., 1998, Buckley et al., 2013). Another line of evidence that argues against the notion that Pard3 is required for C-division is that other intermediate proteins, including aPKC, Cbr1, Crb2a, Nok, and Lin7c, are all absent during C-division (Figures 1 and S1; Yang et al., 2009). Because these intermediate proteins work together with Pard3 (Pocha and Knust, 2013), it seems difficult to suggest that Pard3 localizes and functions alone during C-division, whereas its necessary partners do not show up until P-division stage.

What regulates the three-step apical localization and enrichment of polarity proteins and consequently, the dynamics of apical adhesions, which underlie the very foundation of mirror symmetry formation? Apical localization of polarity proteins may involve many molecular processes, including but not limited to passive diffusion, active transportation, the timing of transcription, the timing of translation, sequential recruitment of proteins through physical binding, and selective degradation by endocytosis. In this study, we show that endocytosis plays a role in OAA dissolution, both N-Cad and ZO-1 undergo basal-to-apical translocation, and Nok and Pard6 play a role in the apical enrichment of Na^+^/K^+^-ATPase. Previously, Buckley et al. reported that N-Cad was enriched in the interdigitating zone in the neural keel; they proposed a plausible model that N-Cad-based interdigitating adhesion may recruit the centrosomes to the midline region, midway along the length of a cell, during early neurulation; the centrosome at the midline region may then organize the mirror-symmetric microtubule assembly, which may regulate polarity protein localization (Buckley et al., 2013). All these findings are probably only revealing the tip of the iceberg of apical protein localization and enrichment. It remains challenging to understand how localization of polarity proteins is coordinated in a strict spatiotemporal order.

### General Application of the Three-Step Process of Tubulogenesis

The three-step zebrafish neurulation may have general implication because tubulogenesis of the gut, pronephric ducts, and pronephric tubules also go through a rod-tube transition (Wallace and Pack, 2003, Ng et al., 2005, Kimmel et al., 1995, Drummond et al., 1998). For example, we observed that during gut development, OAAs and PAAs also first stick gut epithelial cells together into a solid rod and that the opposing adhesions dissolve later to permit the gut lumen to merge; more impressively, the OAAs of the gut rod reside on apical membrane protrusions, which interlock together like hooks to secure the rod-shaped cytoarchitecture (Figures S7I–S7K). In agreement with this general implication, aPKC intermediate apical polarity proteins are required for the tubulogenesis of both the gut and the pronephros (Horne-Badovinac et al., 2001, Gerlach and Wingert, 2014). Thus it is tempting to speculate that opposing adhesions play broad roles in organizing a variety of mirror-symmetric tissues, although the exact molecular compositions of such adhesions may vary from tissue to tissue.

If we strip away the superficial characteristics of symmetry generation during zebrafish neurulation, what would remain are the three core elements: asymmetric individuals (neuroepithelial cells), a symmetric collective (the neural rod or the neural tube), and opposing configuration (via apical adhesions). Here asymmetry and symmetry represent opposite concepts, and yet they depend on each other for their very existence; the interdependency and yet opposition between asymmetry and symmetry is reconciled through apical adhesions, which on the one hand, establish the asymmetry of individual cells, and on the other hand, configure asymmetric individual cells into a symmetric collective. (Interestingly, please note that each asymmetric neuroepithelial cell also displays its own symmetry with its axis of symmetry coinciding with its apicobasal axis.) Such a reconciling process needs to be quantitatively regulated on the levels of both time and space by following the three-step order of expression and localization of polarity proteins. If this stepwise order is not followed, severe disruption of neurulation may occur. For example, precocious expression of Lin7c by mRNA injection resulted in multiple neural tubes in treated embryos (Yang et al., 2009).

The intertwining relationship between symmetry and asymmetry goes beyond tissue formation and can be applied universally at hierarchical levels: all symmetric entities are collectives that are composed of opposingly configured asymmetric components, which themselves display a lower level symmetry, whether perfect or not (Figure 7G). Also, the opposing configuration of asymmetric components can be achieved by a variety of means, such as apical cell-cell adhesions in zebrafish neurulation and gravitation in planet formation.

### Conclusion

Our study reveals a hierarchical framework for zebrafish neurulation: pioneer, intermediate, and terminal polarity proteins enrich and localize apically in a three-step order to orchestrate the formation of a mirror-symmetric neural rod and then a neural tube by dynamically regulating the PAAs and OAAs. This framework implies a general principle that may govern the genesis of many mirror-symmetric tissues: tissue symmetry can be established by organizing asymmetric cells in opposing configurations via polarized adhesions, just like a magician's double-headed coin can be made by gluing two coins together tail to tail. By this simple and yet robust mechanism, the yin-yang duality of tissue symmetry and cellular asymmetry can be reconciled to become an inseparable oneness—asymmetry constructs symmetry and symmetry accommodates asymmetry.

## Methods

All methods can be found in the accompanying Transparent Methods supplemental file.

## Supplemental Information

Document S1. Transparent Methods and Figures S1–S7

Video S1. 3D Presentation of the Distributions of N-Cad^wt^-GFP and ZO-1 in the Neural Keel at 5-ss, Related to Figure 4 Note that ZO-1 foci (red) are scattered throughout the tissue and N-Cad^wt^-GFP (green) are localized broadly on the cell membranes, showing no apparent midline enrichment at this stage. N-Cad^wt^-GFP was induced in *Tg(HSP70:N-Cad*^*wt*^*-GFP)*^*pt137*^ embryos by a 30-min heat shock at 1-ss, and ZO-1 was visualized by immunostaining. This 3D video was made by rotating a series of confocal transverse scans (at 0.3-μm intervals) of the neural keel. The dimensions of the scanned tissue area are 35 μm × 35 μm x 21.9 μm. The white arrowhead points to the midline.

Video S2. 3D Presentation of the Distributions of N-Cad^wt^-GFP and ZO-1 in the Neural Keel at 8-ss, Related to Figure 4 Note that ZO-1 foci (red) and N-Cad^wt^-GFP (green) became enriched in the midline region (arrowhead); however, they had not yet formed circumferential belts at the apical ends of the cells. N-Cad^wt^-GFP was induced in *Tg(HSP70:N-Cad*^*wt*^*-GFP)*^*pt137*^ embryos by a 30-min heat shock at 1-ss, and ZO-1 was visualized by immunostaining. This 3D video was made by rotating a series of confocal transverse scans (at 0.3-μm intervals) of the neural keel. The dimensions of the scanned tissue area are 35 μm × 35 μm x 20.4 μm.

Video S3. 3D Presentation of the Distributions of Both N-Cad^wt^-GFP and ZO-1 in the Early Neural Rod at 14-ss, Related to Figure 4 Note that ZO-1 foci (red) and N-Cad^wt^-GFP (green) became enriched in a midline region (arrowhead) narrower than at 8-ss (Video 2). N-Cad^wt^-GFP was induced in *Tg(HSP70:N-Cad*^*wt*^*-GFP)*^*pt137*^ embryos by a 30-min heat shock at 1-ss, and ZO-1 was visualized by immunostaining. This 3D video was made by rotating a series of confocal transverse scans (at 0.3 μm intervals) of the neural keel. The dimensions of the scanned tissue area are 35 μm × 35 μm x 17.4 μm.

Video S4. Live Imaging of N-Cad^wt^-GFP's Dynamic Distribution in the Hindbrain's Rhombomeres 5 and 6 between 8-ss and 14-ss, Related to Figure 4 N-Cad^wt^-GFP signals (green) were collected at 1-min intervals in the *Tg(HSP70:N-Cad*^*wt*^*-GFP)*^*pt137*^*/ Tg(HSP70: ZO-1.1-mCherry)*^*pt117b*^ double transgenic embryos; the live image frames were then compiled into the video. Note that N-Cad^wt^-GFP signals moved along the cell membranes and eventually enriched at the apical ends of the cells. The dimensions of the images are 105 μm × 105 μm.

Video S5. Live Imaging of ZO-1-mCherry's Dynamic Distribution in the Hindbrain's Rhombomeres 5 and 6 between 8-ss and 14-ss, Related to Figure 4 ZO-1-mCherry (red) were collected at 1-min intervals in the *Tg(HSP70:N-Cad*^*wt*^*-GFP)*^*pt137*^*/ Tg(HSP70: ZO-1.1-mCherry)*^*pt117b*^ double transgenic embryos; the live image frames were then compiled into the video. Note that ZO-1-mCherry positive dots generally moved toward the apical ends, although they often traveled briefly in the basal direction before resuming apical movements. The dimensions of the images are 105 μm × 105 μm.

Video S6. Live Imaging of the Dynamic Distributions of Both N-Cad^wt^-GFP and ZO-1-mCherry in the Hindbrain's Rhombomeres 5 and 6 between 8-ss and 14-ss, Related to Figure 4 The signals of both N-Cad^wt^-GFP (green) and ZO-1-mCherry (red) were collected at 1-min intervals in the *Tg(HSP70:N-Cad*^*wt*^*-GFP)*^*pt137*^*/ Tg(HSP70: ZO-1.1-mCherry)*^*pt117b*^ double transgenic embryos; the live image frames were then compiled into the video. Note that ZO-1-mCherry positive sites became more tightly associated with N-Cad^wt^-GFP over time, displaying increased signal overlapping at the midline region. The dimensions of the images are 105 μm × 105 μm.

Video S7. Live Imaging of the Dynamic Distribution of Both N-Cad^wt^-GFP and ZO-1-mCherry in the Hindbrain's Rhombomeres 5 and 6 between 14-ss and 16-ss, Related to Figure 4 The signals of both N-Cad^wt^-GFP (green) and ZO-1-mCherry (red) were collected at 1-min intervals in the *Tg(HSP70:N-Cad*^*wt*^*-GFP)*^*pt137*^*/ Tg(HSP70: ZO-1.1-mCherry)*^*pt117b*^ double transgenic embryos; the live image frames were then compiled into the video. Note that N-Cad^wt^-GFP signals and ZO-1-mCherry signals initially aligned in a single jaggy line; later, left and right PAAs segregated to reside in two midline-flanking planes. The dimensions of the images are 52 μm × 52 μm.
