# Supplementary material for: Apical Cell-Cell Adhesions Reconcile Symmetry and Asymmetry in Zebrafish Neurulation
Source: iScience. 2018 Apr 13;3:63–85. doi: 10.1016/j.isci.2018.04.007 (PMC5994761; doi:10.1016/j.isci.2018.04.007)
Supplement: Document S1. Transparent Methods and Figures S1–S7 [file mmc1.pdf]

ISCI, Volume *3*

Supplemental Information

Apical Cell-Cell Adhesions

Reconcile Symmetry and Asymmetry

in Zebrafish Neurulation

Chuanyu Guo, Jian Zou, Yi Wen, Wei Fang, Donna Beer Stolz, Ming Sun, and Xiangyun Wei

Supplementary Figures

Figure S1 (related to Figure 1)

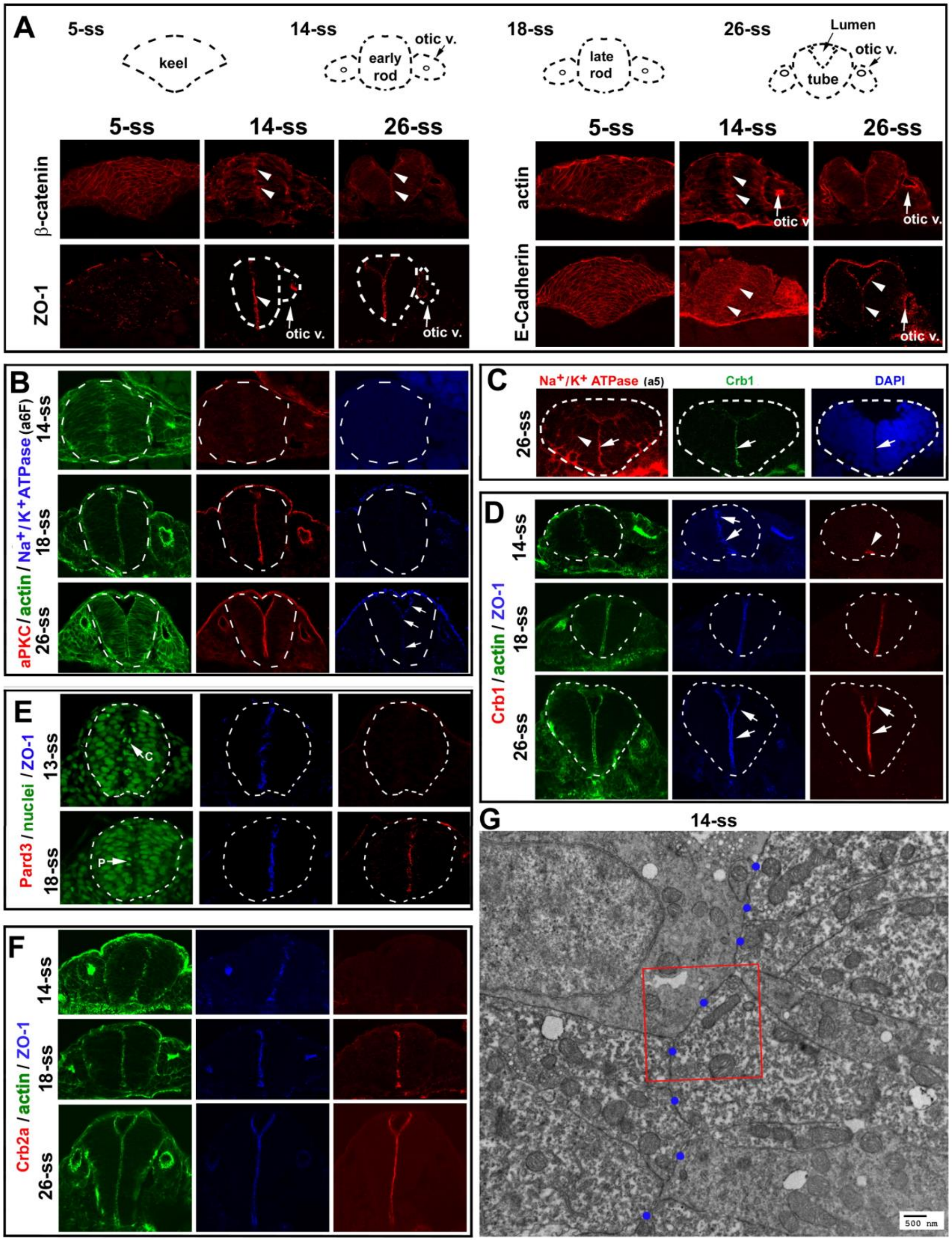

Apical polarity proteins localize or enrich in a stepwise fashion at the apical surfaces in rhombomeres 5 and 6 during neurulation

A. As revealed by immunohistochemistry, E-Cadherin, β-catenin, and F-actin localized broadly to the entire cell membranes at 5-ss, and then enriched apically at 14-ss and 26-ss. At 26-ss, E-

Cadherin signals in the dorsal region of the neural tube and the otic vesicle were stronger than those in the mid-ventral regions. ZO-1 initially scattered as small punctate sites at 5-ss and later enriched apically (14-ss, 26-ss). Arrowheads point at the apical surfaces; arrows otic v. point at the otic vesicles. Note that it is unknown to what extent the E-Cadherin immunostaining signals represented P-Cadherin because the E-Cadherin antibody cross-reacts with P-cadherin (http://www.bdbiosciences.com/ds/pm/tds/610182.pdf). **B**. Simultaneous immunostaining of F-actin bundles, aPKC, and Na^+/K^+ ATPase (with the a6F antibody) at 14-ss, 18-ss, and 26-ss. Note the lack of aPKC staining at 14-ss as well as Na^+/K^+ ATPase staining at 14-ss and at 18-ss. **C**. Apical enrichment of Na^+/K^+ ATPase α (with weak lateral membrane staining) at 26-ss was also confirmed with the a5 antibody. Note that all other Na^+/K^+ ATPase α images in this study were visualized with the a6F antibody. **D**. Simultaneous immunostaining of ZO-1, F-actin, and Crb1 at 14-ss, 18-ss, and 26-ss. Note the lack of Crb1 staining at 14-ss, except at the very ventral end (arrowhead). **E**. Simultaneous immunostaining of ZO-1 and Pard3, counterstained with nuclear dye DAPI (blue). Note the lack of Pard3 signals at 13-ss. Arrow C indicates a cell going through a C-division at 13-ss when Pard3 was not detectable; arrow P indicates a cell going through a P-division at 18-ss when Pard3 became detectable at the apical surfaces. **F.** Simultaneous immunostaining of F-actin bundles, ZO-1, and Crb2a. Note the lack of Crb2a at 14-ss. **G**. A TEM image at a lower magnification illustrates the apical surfaces (marked by blue dots) juxtaposed at the midline. The boxed area was magnified in Figure 1E.

Figure S2 (related to Figure 2)

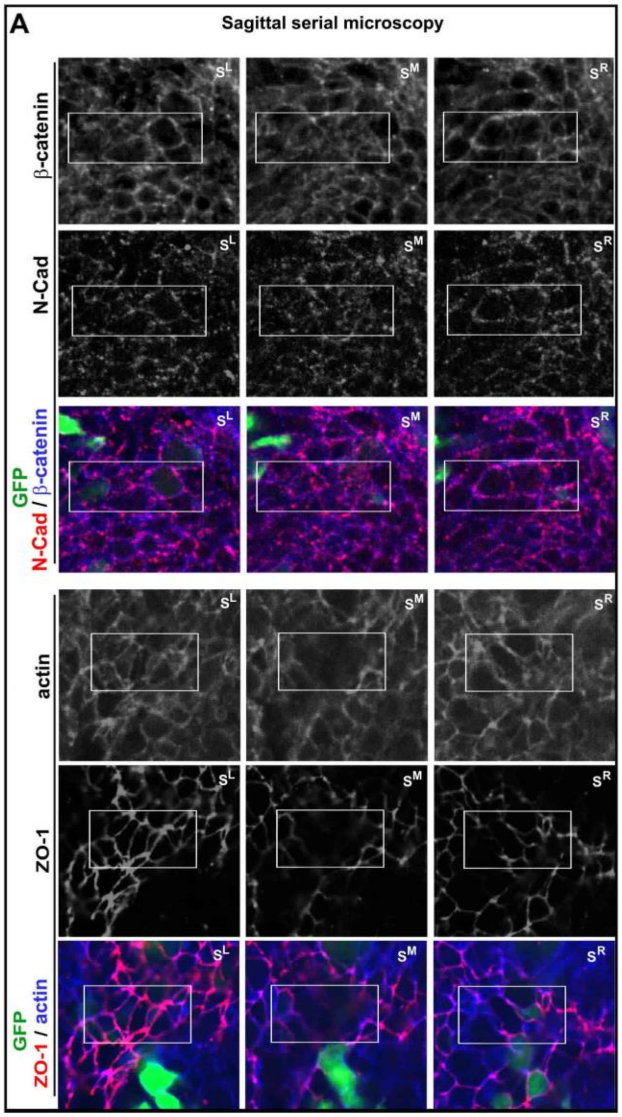

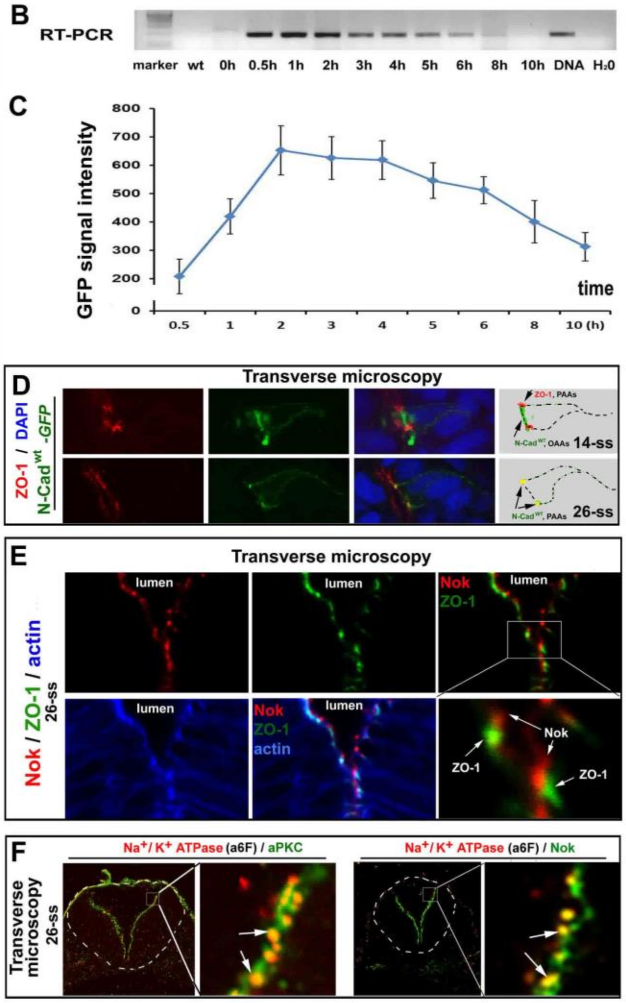

G Distribution of immuno-gold particles in N-Cad-GFP transgenic or non-transgenic fish

	total gold particles	@ PAAs	@ OAAs	@ apical surfaces	@ cytoplasm
transgenic N-Cad-GFP^wt (205 cells)	494	189 (38%)	142 (29%)	73 (15%)	90 (18%)
non-transgenic control (206 cells)	21	2 (9.5%)	0	0	19 (90.5%)

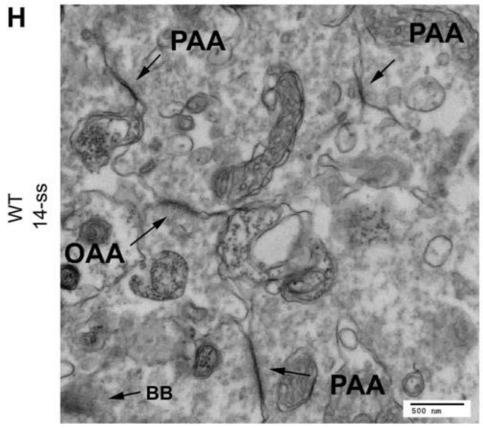

The development of the PAAs and OAAs and their dynamic molecular compositions

A. GFP-assisted sagittal serial microscopy revealed that N-Cad and β-catenin but not ZO-1 or actin localized to the opposing apical surfaces. The boxed areas are presented in Fig. 2A at higher magnifications. **B.** An RT-PCR revealed that the mRNA products of the *N-Cad^{wt}-GFP* transgene reached its highest level 30 min after a heat shock and disappeared completely by 10 hours after the heat shock. **C**. The N-Cad^{wt}-GFP fluorescence intensities of whole embryos reached the highest level 2 hours after the heat shock, and fell by 77% 10 hours after the heat shock. The fluorescence intensities were measured with Q-imaging under a stereo-fluorescence scope (ten embryos; means ± SEM). **D**. Single channel and merged images of Fig. 2B are presented to better illustrate the distributions of N-Cad^{wt}-GFP to the PAAs and OAAs; N-Cad^{wt}-

GFP was transiently-induced by heat shock from a transgenic construct injected into the embryos. Note that N-Cad^{wt}-GFP enriched at both the PAAs and OAAs at 14-ss, but only at the PAAs at 26-ss. Weaker N-Cad^{wt}-GFP signals persisted on the lateral membranes. The drawings summarize the distributional dynamics of ZO-1 and N-Cad-GFP. **E.** Nok, a cytoplasmic partner of Crb proteins, closely associated with the apical side of ZO-1 sites. **F**. Na+/K+ ATPase α colocalized with aPKC and Nok at 26 ss (arrows), although Nok and aPKC distributed more broadly than Na+/K+ ATPase α did. **G**. The table presents the subcellular distributions of immuno-EM gold particles in the early neural rods (at 14-ss) of N-Cad-GFP^{wt}-expressing transgenic embryos and non-transgenic wildtype control embryos: The numbers of gold particles (as well as their corresponding percentages in total particles) distributed at the PAAs, OAAs, opposing apical surface, and cytoplasm are tabulated. Note the enrichment of gold particles at the OAAs and PAAs in N-Cad-GFP^{wt}-expressing embryos but not in wildtype control embryos. **H**. An immuno-EM micrograph of the midline region of a wildtype neural rod revealed that the PAAs and OAAs survived the Triton X-100 extraction of the staining procedure and that no gold particles were observed at the PAAs and OAAs.

Figure S3 (related to Figure 3)

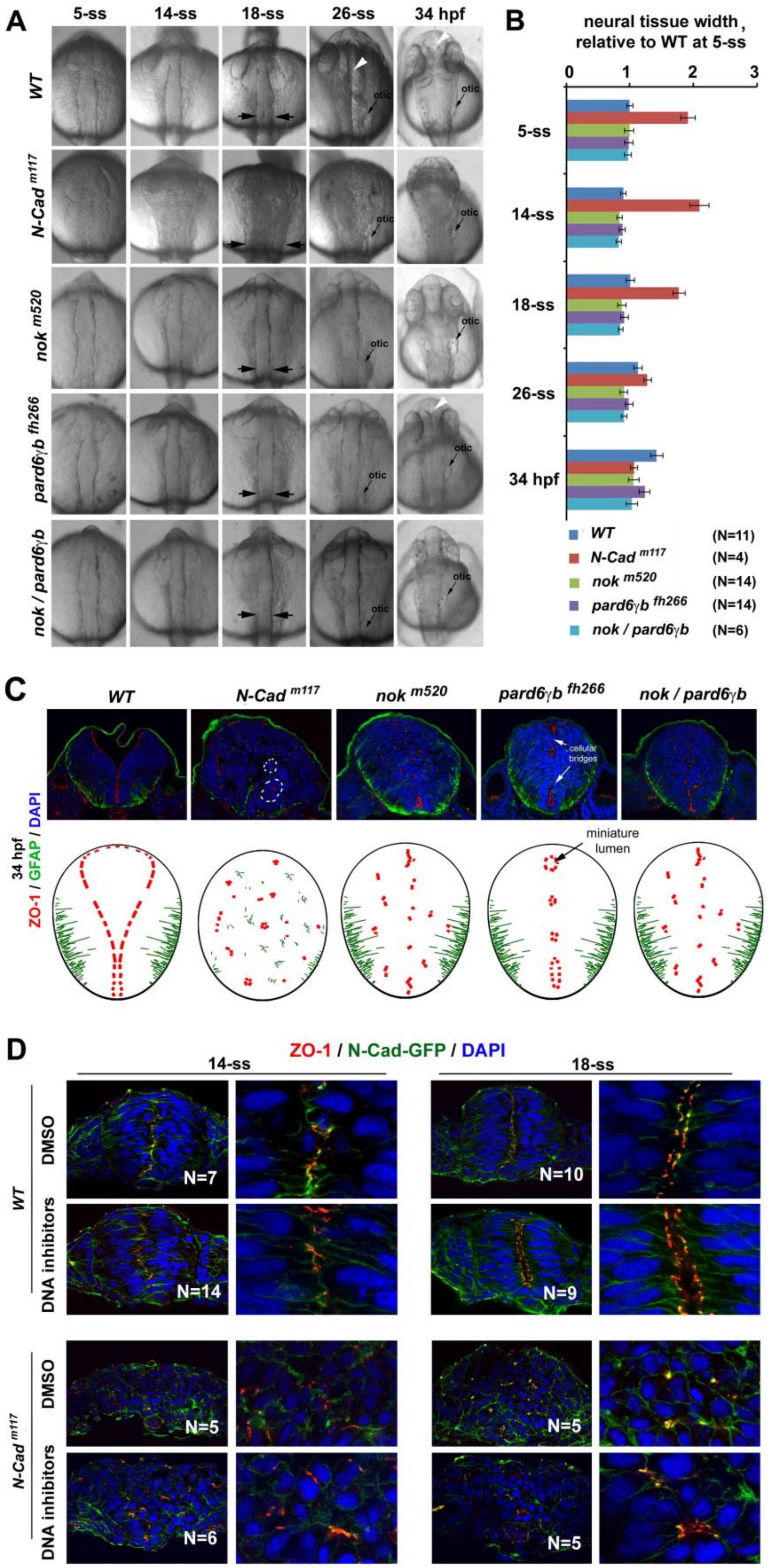

Loss of pioneer protein N-Cad and intermediate proteins Nok and Pard6γb prevented neural tube lumen formation and affected the mirror symmetry of the neural tissue

A. Dorsal views of the neural tissues of wildtype, *N-Cad*^{m117}, *nok*^{m520}, *pard6γb*^{fh266}, and *nok*^{m520}/*pard6γb*^{fh266} embryos during neurulation. At 26-ss and 34 hpf (hours post-fertilization),

the lumen was apparent in wildtype (white arrowhead) but not in mutants. **B**. Bar graphs illustrate that the neural tissue of *N-Cad*^{m117} mutants was 170% to 200% wider than wildtype and other mutants (measured from 5-ss to 18-ss at the otic vesicle regions marked by the opposing black arrows in A; means ± SEM; N numbers, measured embryos). **C**. At 34 hpf, apical marker ZO-1 localized ectopically in *nok*^{m520} single (6 embryos) and *nok*^{m520}/*pard6γb*^{fh266} double mutations (7 embryos), although the basal marker GFAP appeared to localize properly; in *pard6γb*^{fh266} (6 embryos), apical marker ZO-1 continued localizing to the midline region and demarcated the boundaries of miniature lumens (black arrow); finally, in *N-Cad*^{m117} (20 embryos), the mirror symmetry of the neural tissue remained disrupted at 34 hpf, and cells aggregated into rosettes (two rosettes are marked with dashed lines). The drawings summarize the distributions of the apical and basal markers at 34 hpf. **D**. Effects of inhibiting cell division with DNA synthesis inhibitors (from 1-ss to 18-ss) on the distribution of apical markers ZO-1 and N-Cad-GPF in wildtype and *N-Cad*^{m117} at 18-ss. Note that in wildtype, apical markers localized in a jaggy line at the midline region at 14-ss and then separated into two parallel lines at 18-ss; by contrast, in *N-Cad*^{m117}, apical markers scattered throughout the tissue at both 14-ss and 18-ss, whether treated with the inhibitors or not. N numbers are numbers of embryos examined for each condition. Left panels are lower magnifications, right panels are higher magnifications.

Figure 4S (related to Figure 4)

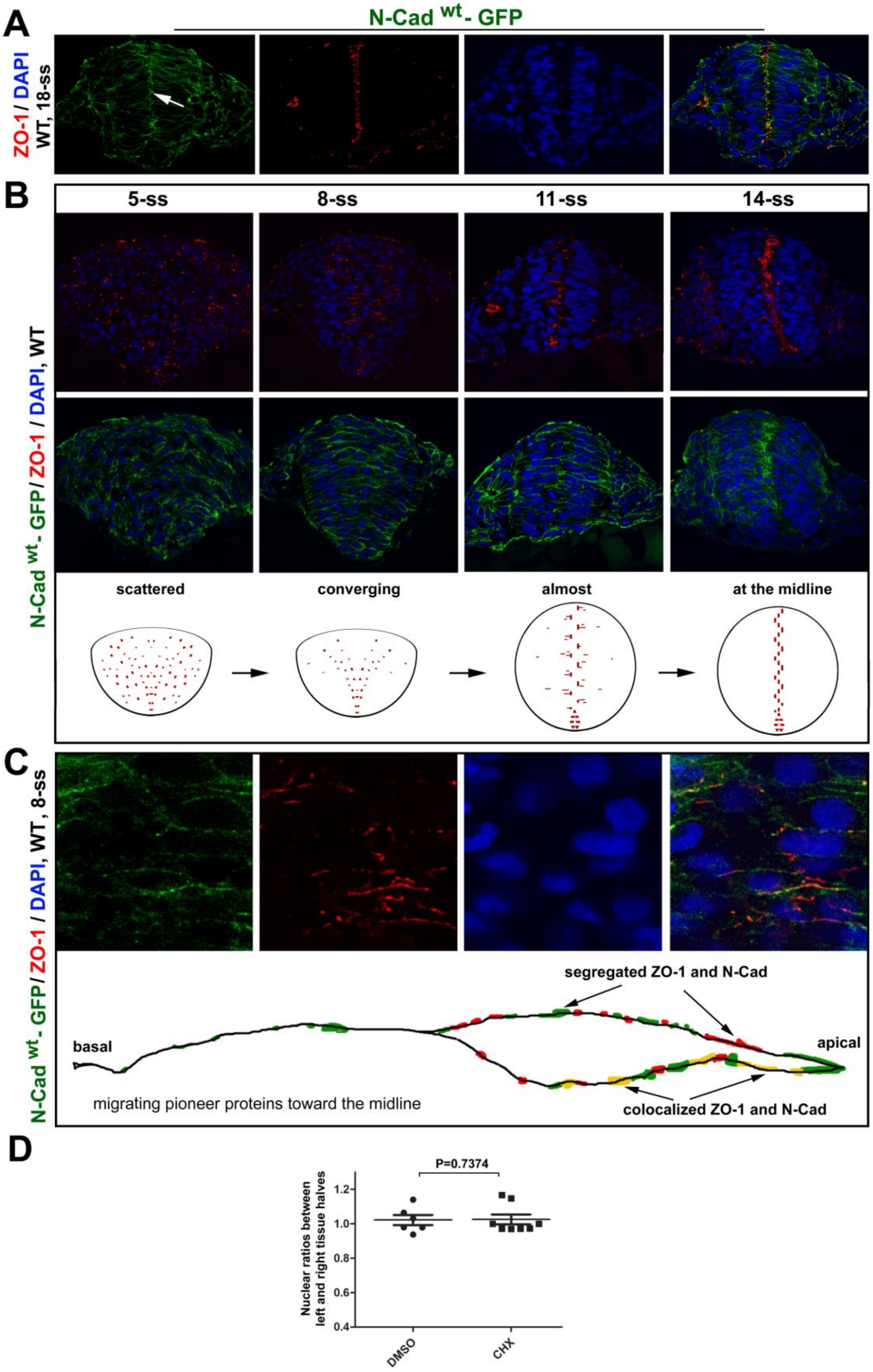

ZO-1 and N-Cad^{wt}-GFP translocate apically during neural keel-neural rod transition in *Tg(HSP70:N-Cad^{wt}-GFP)^{pt137}*

A. Distributions of N-Cad^{wt}-GFP and ZO-1 in the neural rod at 18-ss were revealed by immunohistochemistry of the transverse tissue sections. Note the apical enrichment of both

proteins (arrows). N-Cad^{wt}-GFP was briefly induced by a heat shock at 10-ss. **B**. Transverse imaging at 5-ss, 8-ss, 11-ss, and 14-ss: ZO-1 first localized to small punctate sites and then enriched at the midline region, whereas N-Cad^{wt}-GFP distributed broadly on the cell membranes and then enriched at the midline region. Diagrams summarize the dynamics of ZO-1 distributions. **C**. A higher magnification to better reveal that ZO-1 foci, which were often closely associated with N-Cad^{wt}-GFP, became more concentrated in the apical half of the cells at 8-ss and localized to larger and more elongated spots than at earlier stages. The bottom diagram depicts the distribution of ZO-1 and N-Cad^{wt}-GFP on the cell membrane. **D**. Individual-value bar graphs (means ± SEM) show that the cycloheximide (CHX) treatment (see the protocol in Fig. 4E) did not affect the nuclear ratios between the left and right halves of the neural tissue. Numbers of embryos analyzed: 6 DMSO-treated; 8 CHX-treated. *P* values by two-tailed *t* test.

Figure S5 (related to Figure 5)

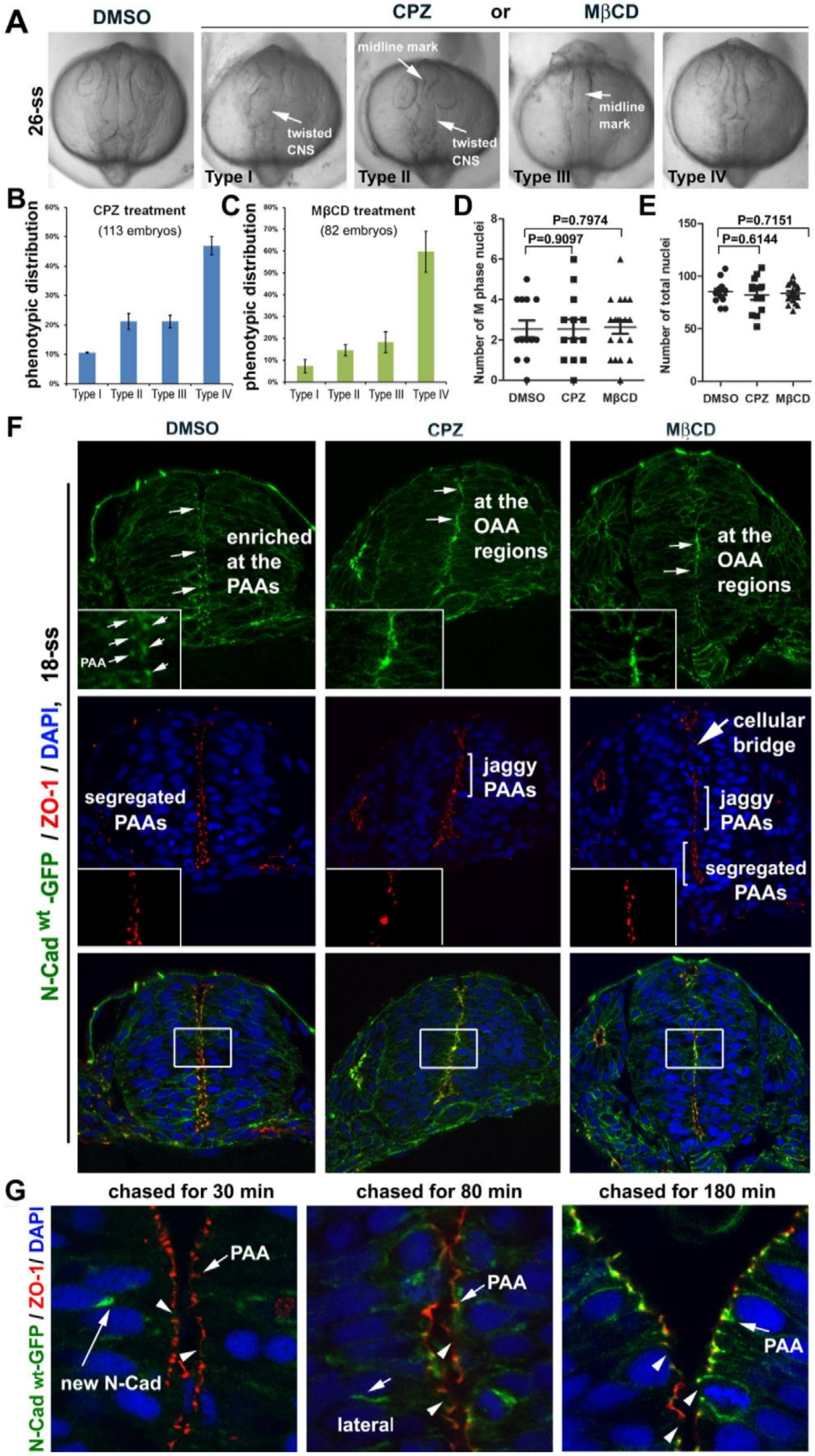

Endocytosis is required for OAA dissolution

A-C. Endocytosis inhibitors CPZ and MβCD blocked lumen formation and caused the neural tissue to twist to various degrees, as classified into Type I – IV categories. The cause of the

phenotypic variations is unclear, but we speculate that embryos responded to the drugs differently due to variations in their genetic background, despite the fact that they were all TU fish. Histograms represent the frequency distribution of the four categories (from three experiments of a total of 113 embryos treated with PCZ, 82 embryos treated with MβCD, and 50 control embryos treated with DMSO; means ± SEM). **D.** The average numbers of dividing nuclei per section (by counting M-phase nuclei revealed by DAPI staining) did not vary among embryos treated with DMSO (12 embryos), CPZ (13 embryos), and MβCD (20 embryos). **E.** The average total numbers of nuclei per section did not vary among embryos treated with DMSO (12 embryos), CPZ (13 embryos), and MβCD (20 embryos). **F**. The effects of endocytosis inhibitors CPZ and MβCD (from 11-ss to 18-ss) on the apical adhesions in *Tg(HSP70:N-Cad^{wt}-GFP)^{pt137}* embryos at 18-ss. Note that the inhibition of endocytosis resulted in jaggy midline alignment of ZO-1 and N-Cad-GFP, which were sometimes disrupted by cellular bridges; however, in DMSO controls, the PAAs segregated into two parallel planes flanking the midline, where the OAAs dissolved. Insets, magnifications of the boxed regions in the top and middle panels; the boxed regions in the bottom panels were presented at higher magnifications in Figure 5A. **G**. The images show the tissue regions from which local apical areas were magnified and presented in Figure 5G, which illustrates the fate of N-Cad^{wt}-GFP that was briefly induced at 18-ss. Again, note that N-Cad^{wt}-GFP first concentrated in some intracellular regions (30 min), then localized to the lateral membrane (80 min), and finally enriched at the PAAs (arrows; 180 min); however, at no time did N-Cad^{wt}-GFP localize to the OAA regions (arrowheads).

Figure S6 (related to Figure 6)

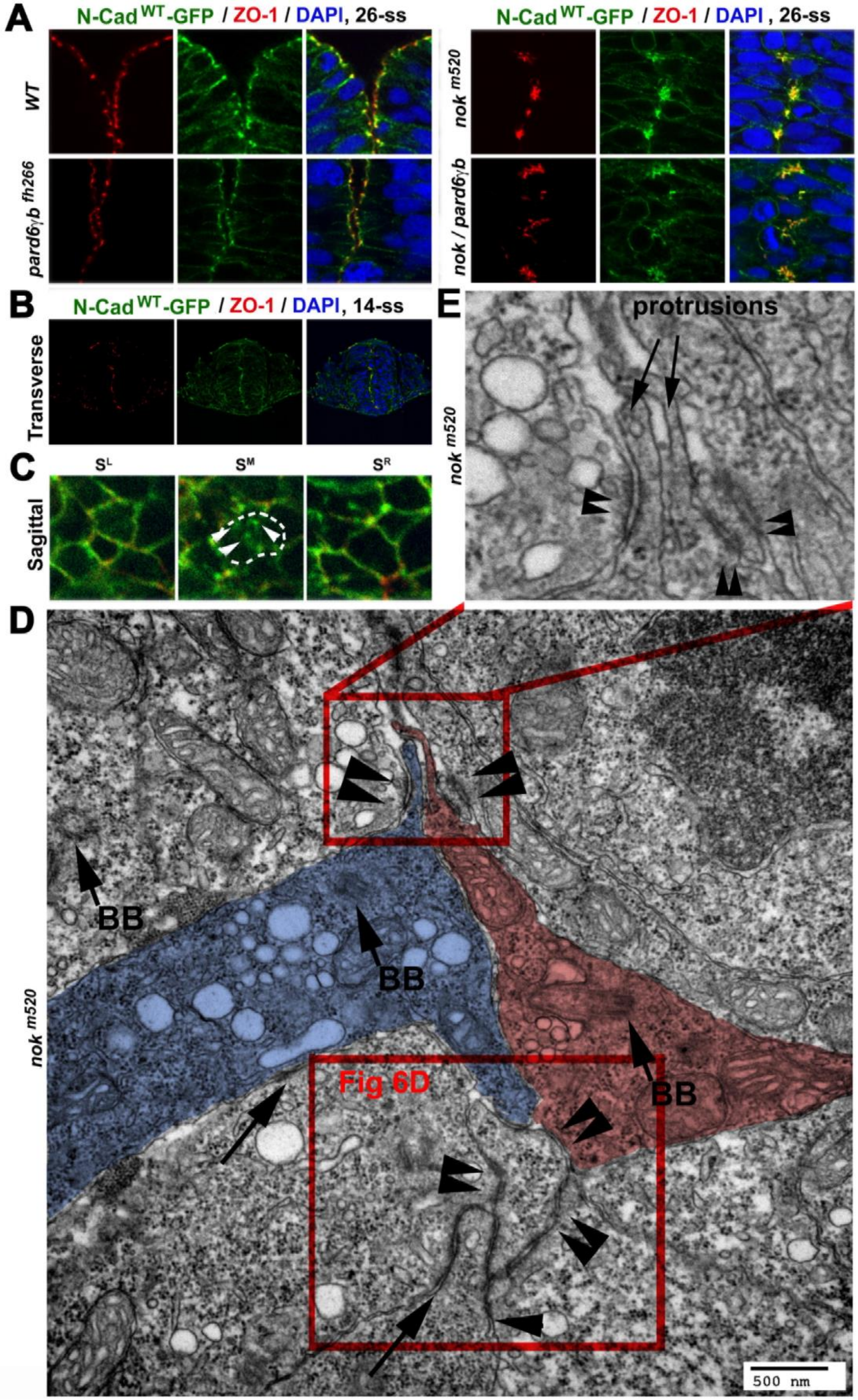

Intermediate proteins maintain PAA integrity and specificity

A. Single channel images of Figure 6A illustrate the distribution of ZO-1 and N-Cad^{wt}-GFP in wildtype, *nok*^{m520}, *pard6γb*^{fh266}, and *nok*^{m520}/*pard6γb*^{fh266} mutant embryos at 26-ss. **B**. Transverse

confocal imaging of 21 siblings of *nok*^{m520} heterozygous parents revealed no difference in the apical distributions of ZO-1 and N-Cad-GFP at 14-ss. (Please note that unlike at 18-ss or later, *nok*^{m520}mutants do not display apparent phenotypes at 14-ss for easy genotyping. Thus, we decided to infer whether *nok* mutation affects the OAAs and PAAs at 14-ss by examining over 20 embryos, of which about five embryos were expected to be *nok*^{m520} mutants.) **C**. Sagittal confocal imaging of 22 siblings of *nok*^{m520} heterozygous parents confirmed the punctate N-Cad-GFP-positive OAAs (arrowheads) in the midline plane (S^M) at 14-ss in all embryos examined. **D**, **E**. A TEM image shows the distributions of cell-cell adhesions at the midline region. Cell-cell adhesions assembled cell protrusions in plywood-like clusters (double arrowheads). Two neuroepithelial cells, highlighted with blue and magenta, opposed at their apical ends, which are indicated by the apical marker basal bodies (arrows BB). The lower boxed region in B is magnified in Figure 6D; the upper boxed region is enlarged in C to better illustrate the plywood-like clustering of cell protrusions that were adhered together by cell-cell adhesions (double arrowheads).

Figure S7 (related to Figure 7)

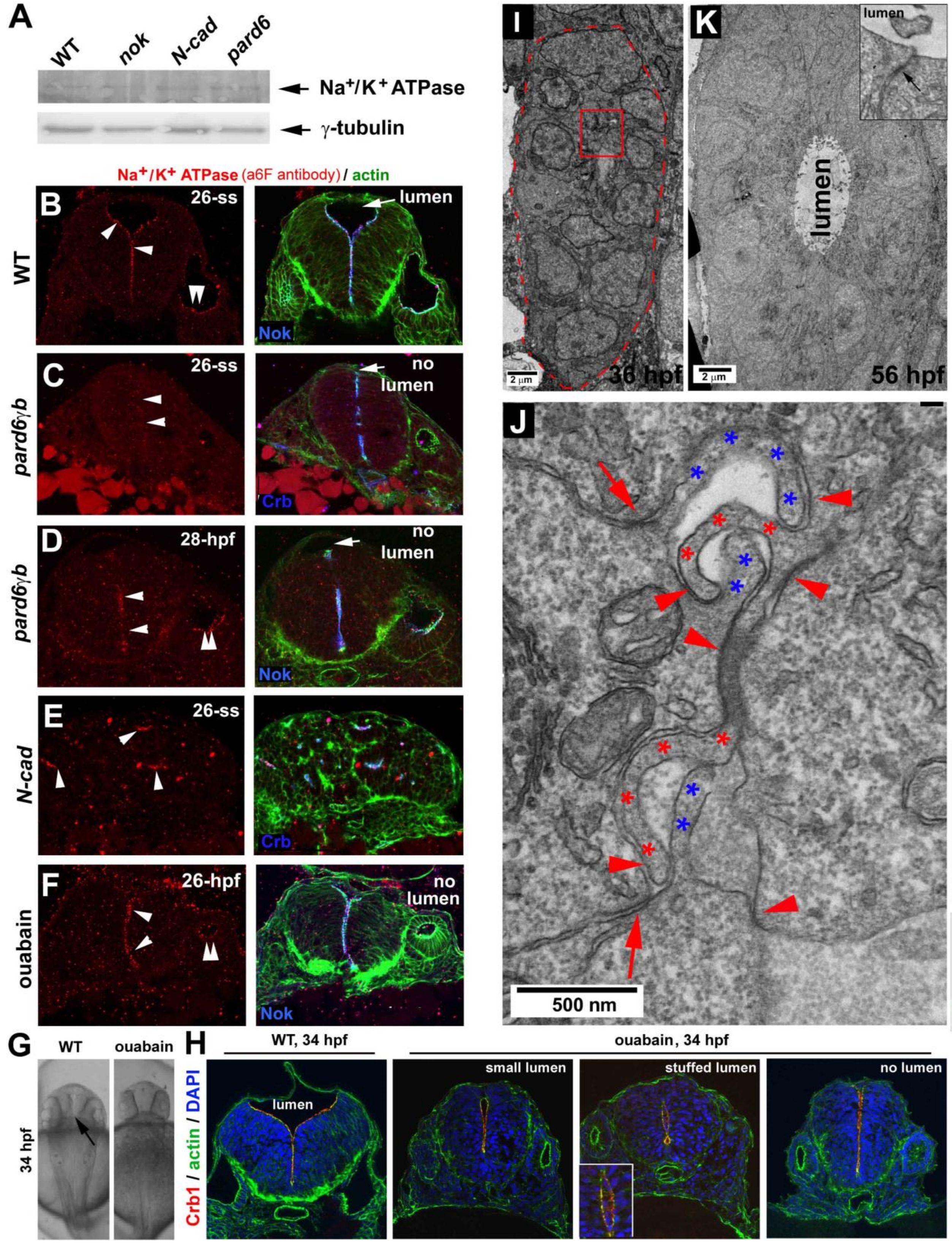

Terminal protein Na⁺/K⁺ ATPase is required to inflate the neural tube lumen, and the gut development also follows a rod-tube transition

A. Western blotting revealed that Na^+/K^+ ATPase α1 was expressed at 26-ss in *nok*^{m520}, *N-Cad*, and *pard6γb*^{fh266} mutants as in wildtype. **B.** Na^+/K^+ ATPase α co-localized with Nok at the apical surfaces in wildtype at 26-ss. **C-D**. Na^+/K^+ ATPase α localized apically in *pard6γb*^{fh266} mutants

at 28 hpf but not yet at 26-ss. **E**. In *N-Cad* mutants, Na^+/K^+ ATPase α localized to the central miniature lumen of neural cellular rosettes at 26-ss. **F**. Ouabain treatment did not prevent Na^+/K^+ ATPase α from localizing apically but did prevent the lumen from inflating. **G.** Dorsal view of the brain ventricles (arrow) in 34-hpf wildtype but not in ouabain-treated embryos. **H.** Ouabain treatment from 1-ss to 34 hpf: Crb1 and actin localized normally at the apical ends of the neural tubes (transverse sections) in wildtype (9 embryos) and in ouabain-treated embryos (30 embryos); however, ouabain-induced three defects in the lumen: small lumen (6 out of 30), stuffed lumen (stuffed with cells, inset; 4 out of 30), and no lumen (20 out of 30). **I**. At 36 hpf, the gut epithelial cells organized into a rod. The red-dashed line demarcates the outer boundary of the gut rod. **J**. A boxed region in panel I is enlarged to better show the hook-like interlocking apical membrane protrusions of gut rod cells (red and blue asterisks). These hook-like apical protrusions are presumably the microvilli, and they were adhered together with adhesions (arrowheads) to join opposing cells together. In addition, the parallel apical adhesion complexes (arrows) adhered together neighboring cells of the same orientations. **K**. At 56 hpf, the gut tissue developed into a hollow tube. Microvilli were no longer interlocked with each other via opposing adhesions, and a lumen emerged. However, the parallel apical adhesion persisted (inset, arrow).

Transparent Methods

Zebrafish care and mutant polarity gene fish lines

Tubingen (TU) wildtype, *nok*^{m520} (Wei and Malicki, 2002), *N-Cad*^{m117} (Malicki et al., 2003), *pard6γb*^{fh266} (Grant and Moens, 2010), and transgenic fish were maintained on a 14-hr light/10-hr dark cycle. Zebrafish embryos were raised at 28.5°C. Animal care and handling conformed to University of Pittsburgh guidelines. The *nok*^{m520} and *N-Cad*^{m117} mutations most likely cause null phenotypes in neurulation because their functions are unlikely rescued by their paralogs: *nok's* closest paralog *ponli* is restrictively expressed in the retina, making *nok* an essential gene for neural tube development (Zou et al., 2010; Fang et al., 2017). *N-Cad*'s closest paralog in zebrafish is per a BLAST search; however, R-Cad is not expressed in the neural tube (Liu et al., et al., 2003). Among four *pard6* paralogues, *pard6α*, *pard6β*, *pard6γa*, and *pard6γb*, only *pard6α* mRNA could partially rescue *pard6γb* morphant phenotypes; however, morpholino suppression of *pard6α* could not phenocopy *pard6γb* morphant phenotypes, suggesting that *pard6γb* plays important roles in neurulation (Munson et al., 2008).

Generation of transgenic fish

The heat-shock-inducible *Tg(HSP70:N-Cad*^{wt}*-GFP)*^{pt137}, *Tg(HSP70:N-Cad*^{m117}*-GFP)*^{pt136}, and *Tg(HSP70: ZO-1.1-mCherry)*^{pt117b} transgenic fish lines (ZO-1.1 Genebank number: KY794711) were generated with the I-SceI meganuclease-based transgenic system (Thermes et al., 2002; vector from Michael Tsang). 20 pg of each construct were injected into 1-cell embryos along with 0.01U of I-SceI. The injected embryos were heat-shocked at 37ºC for 1 hour and screened 2 hours later for GFP or mCherry expression; positive fish were raised as founder fish, which were outcrossed with TU wildtype fish to select for stable transgenic lines.

Genotyping mutant embryos

N-Cad^{m117} mutants were genotyped with the dCAPs method (derived Cleaved Amplified Polymorphic Sequence; Michaels and Amasino, 1998). Specifically, we used a forward primer (5'ctgaatcgcgtgaacagagac3') and a reverse primer (5'ccacacatttctgactgcac3') to amplify a 152-bp *N-Cad*^{m117} mutation site-containing a DNA fragment by PCR with the tail DNA as templates. The genotypes of template DNA were determined by the digestibility of the 152-bp DNAs by AlwNI, which cuts the wildtype but not the mutant DNA into a 123-bp and a 19-bp fragment.

To genotype *pard6γb*^{fh266} mutants, we used a forward primer (5'ccattcctgcagtgttggcaaattc3') and a reverse primer (5'aacagcacaaggcaaaactgggtca3') to amplify a 495-bp genomic DNA fragment PCR and then digest it with BsmA I, which cuts the wildtype but not mutant DNA into a 395-bp and a 100-bp fragment (Grant and Moens, 2010).

To genotype *nok*^{m520} mutants, we used a forward primer (5'gtttgctggacctgttcctcgtaag3') and a reverse primer (5'gtctgtgctggtgccgtaaaagttc3') to amplify a 244-bp genomic DNA fragment by PCR and then digest it with Sal I, which cuts the wildtype but not mutant DNA into an 112-bp fragment and a 132-bp fragment (Wei and Malicki, 2002).

Inhibition of DNA synthesis

To assess Nok's role in maintaining the PAAs, we blocked cell division from 10-ss to 26-ss by incubating embryos with DNA synthesis inhibitors aphidicolin (150 μM; Sigma-Aldrich, cat# A0781) and hydroxyurea (20 mM; Sigma-Aldrich, cat# H8627) in 4% DMSO (Sigma-Aldrich, D8418) (Lyons et al., 2005). To assess the role of cell division in mirror symmetry generation, wildtype and *N-Cad*^{m117} mutant embryos were incubated with the DNA synthesis inhibitors from 1-ss to 26-ss.

Inhibition of protein synthesis

To block new protein synthesis so as to follow the fate of N-Cad^{wt}-GFP, *Tg(HSP70:N-Cad*^{wt}*-GFP)*^{pt137}/ *Tg(HSP70: ZO-1.1-mCherry)*^{pt117b} double transgenic embryos were first heat-shocked for 1 hour, starting at 5-ss, to induce fusion proteins, and then, between 8-ss and 18-ss, treated with 100 μg/ml Cycloheximide in 4% DMSO or with 4% DMSO alone (Sigma, C7698; Poulain and Lepage, 2002).

Inhibition of endocytosis

To block endocytosis so as to determine if endocytosis is required for OAAs dissolution, zebrafish embryos were treated, from 11-ss to 18-ss, with 700 μM Chlorpromazine hydrochloride (CPZ) (Sigma-Aldrich, cat# C8138) or with 3 mM Methyl-β-cyclodextrin (MβCD) (Sigma-Aldrich, cat# C4555) in 4% DMSO. To quantitatively evaluate the effects of endocytosis inhibition on OAA dissolution, the midline distribution of N-Cad^{wt}-GFP and ZO-1 at18-ss was classified as either jaggy as in the jaggy early neural rod, or as segregated as in the normal smooth late neural rod (Fig. 5; Fig. 4; Supplemental Fig. 4). The dorsal-ventral span (D-V span) of the neural tissue is defined as the interval between the dorsal apex and ventral bottom of the neural tissue. The D-V spans of jaggy apical marker alignment and cellular bridges were presented as fractions of the total D-V span. To calculate the fractions of cells that displayed N-Cad^{wt}-GFP at the OAA regions in treated 18-ss embryos among the total number of cells examined, consecutive optical sections were examined so as not to mistake PAA signals for the OAA signals.

Inhibition of Na^+/K^+ ATPase

To block the ion-exchanging function of Na^+/K^+ ATPase, the embryos were incubated with 7 mM ouabain (Sigma-Aldrich, cat# O3125) in E3 egg water.

Immunohistochemistry

The following fixation conditions were used for various immunostaining: for E-cadherin, N-cadherin, and β-catenin, embryos were fixed with 4% PFA plus 5% sucrose and 1mM $CaCl_2$ for 30 minutes at RT; for Na+/K+ATPase with a5 antibody, embryos were fixed in Dent's solution (80% methanol and 20% DMSO), followed by 1% SDS treatment (Brown et al., 1996); for all other antigens, embryos were fixed with 4% PFA for 2 hours at RT.

The following rabbit polyclonal antibodies and working dilutions were used: anti-Nok (1:300, Wei and Malicki, 2002), anti-Pard3 (1:50, Wei et al., 2004), anti-Crb1$^{aa952-1258}$, anti-Crb2a$^{aa97-457}$, anti-Crb2b-lf $^{aa466-773}$ (1:300, Zou et al., 2012; Zou et al., 2013), anti-Lin7 (1:300, Wei et al., 2006), anti- PKCζ (1:300; Santa Cruz, cat# sc-216; RRID: AB_2300359), anti-N-Cadherin (gift from Dr. Qin Liu 1:50; Liu et al., 2001). The following mouse monoclonal antibodies and working dilutions were used: anti-ZO-1 (1:300, Life Technologies; cat# 339100; RRID:AB_87181), a6F and a5 anti-Na^+/K^+ ATPase α (1:100; Developmental Studies Hybridoma Bank, a6F for Na^+/K^+ ATPase α1; RRID:AB_528092, and a5 for all Na^+/K^+ ATPase α isoforms; RRID: AB_2166869), anti-E-Cadherin (1:200, BD Transduction Laboratories, cat# 610182; RRID: AB_397581), anti-β-catenin (1:200; Sigma-Aldrich, cat# c7207; RRID: AB_476865). Finally, Alexa-488- or Alexa-647-conjugated Phalloidin (1:200, Invitrogen cat# A12379; RRID: AB_2315147; cat# A22287; RRID: AB_2620155) were used to visualize F-actin. Confocal microscopy was performed with an Olympus Fluoview FV1000 laser scanning microscope.

Sagittal serial microscopy
To reveal en face distribution of polarity proteins in the neural tissue, we devised the GFP-assisted sagittal serial microscopy (Fig. 2A). Specifically, a small number of cells were first highlighted with GFP, which was expressed in a mosaic fashion from an *ef1α*-driven GFP transgenic construct that was injected into the embryos at the 1-cell stage. Utilizing the GFP signals, the opposing apical surfaces as well as the flanking PAA regions can be pinned down to a narrow transitional zone at the midline between GFP-positive cells and GFP-negative cells by serial optical sectioning at 0.3 µm intervals in sagittal planes (Fig. 2A; S^M) (Fig. 2A; S^L and S^R). Sagittal imaging was also employed without GFP assistance in some cases, in which the midline region was identified by the flanking PAA signals and by its location dorsal to the notochord.

Transmission electron microscopy
The samples were fixed with 2% paraformaldehyde plus 2.5% electron microscope (EM)-grade glutaraldehyde in 0.1M PBS (pH 7.3) at RT, rinsed in PBS, post-fixed with 1% OsO_4 and 1% $K_3Fe(CN)_6$, dehydrated through a graded series of ethanol, and embedded in Epon (Polysciences, INC. Warrington, PA, USA). The tissues were sectioned at 65 nm and stained with 2% uranyl acetate and Reynold's lead citrate and then examined with a Jeol 1011 transmission EM.

Immuno-EM
N-Cad-GFP-expressing embryos were treated at 14-ss with 0.5% Triton X-100 in PBS for 1 min, fixed with 2% PFA in PBS in the presence of 0.5% Triton for 1 h, blocked with 20% NGS (Normal Goat Serum) for 1 h, incubated with anti-GFP antibody (1:500 in 20% NGS, Abcam, cat# ab6556) overnight at 4°C, and then incubated with 6 nm Colloidal Gold AffiniPure Goat Anti-Rabbit IgG (1:10 in 20% NGS, Jackson ImmunoResearch Inc, cat# 111-195-144) overnight at 4°C. After 4 X 1 h washes with washing solution, the embryos were fixed again with 2.5% glutaraldehyde for 1 h, and then processed by the TEM procedure.

Western blotting
To examine Na^+/K^+ ATPase α protein level by Western blotting (Supplemental Fig. 7A), 26-ss embryos of various genetic backgrounds were homogenized (yolk removed with forceps) with a lysis buffer (1% Triton X-100 in 1 x PBS, with 1 x cocktail protease inhibitors, Roche, cat# 11245220). Centrifugation supernatants of the lysates from 30 *N-Cad*^{m117} mutants and 20 embryos of other backgrounds were electrophorized on a 10% SDS polyacrylamide gel and then analyzed by Western blotting with a6F anti-Na^+/K^+ ATPase α1 and anti-γ-tubulin antibodies (1:1000, Sigma, cat# T3320).

Supplemental References

Brown, D., Lydon, J., McLaughlin, M., Stuart-Tilley, A., Tyszkowski, R., and Alper, S. (1996). Antigen retrieval in cryostat tissue sections and cultured cells by treatment with sodium dodecyl sulfate (SDS). Histochem Cell Biol 105, 261-267.

Fang, W., Guo, C., and Wei, X. (2017). rainbow enhancers regulate restrictive transcription in teleost green, red, and blue cones. J Neurosci.

Grant, P. K., and Moens, C. B. (2010). The neuroepithelial basement membrane serves as a boundary and a substrate for neuron migration in the zebrafish hindbrain. Neural Dev 5, 9.

Liu, Q., Babb, S. G., Novince, Z. M., Doedens, A. L., Marrs, J., and Raymond, P. A. (2001). Differential expression of cadherin-2 and cadherin-4 in the developing and adult zebrafish visual system. Vis Neurosci 18, 923-933.

Liu, Q., Ensign, R. D., and Azodi, E. (2003). Cadherin-1, -2 and -4 expression in the cranial ganglia and lateral line system of developing zebrafish. Gene Expr Patterns 3, 653-658.

Lyons, D. A., Pogoda, H. M., Voas, M. G., Woods, I. G., Diamond, B., Nix, R., Arana, N., Jacobs, J., and Talbot, W. S. (2005). erbb3 and erbb2 are essential for schwann cell migration and myelination in zebrafish. Curr Biol 15, 513-524.

Michaels, S. D., and Amasino, R. M. (1998). A robust method for detecting single-nucleotide changes as polymorphic markers by PCR. Plant J 14, 381-385.

Poulain, M., and Lepage, T. (2002). Mezzo, a paired-like homeobox protein is an immediate target of Nodal signalling and regulates endoderm specification in zebrafish. Development 129, 4901-4914.

Thermes, V., Grabher, C., Ristoratore, F., Bourrat, F., Choulika, A., Wittbrodt, J., and Joly, J. S. (2002). I-SceI meganuclease mediates highly efficient transgenesis in fish. Mech Dev 118, 91-98.

Zou, J., Yang, X., and Wei, X. (2010). Restricted localization of ponli, a novel zebrafish MAGUK-family protein, to the inner segment interface areas between green, red, and blue cones. Invest Ophthalmol Vis Sci 51, 1738-1746.
